# Improved predictions of time-dependent drug-drug interactions by determination of cytosolic drug concentrations

**DOI:** 10.1038/s41598-019-42051-x

**Published:** 2019-04-10

**Authors:** Anne M. Filppula, Rezvan Parvizi, André Mateus, Pawel Baranczewski, Per Artursson

**Affiliations:** 1 Department of Pharmacy and Uppsala Drug Optimization and Pharmaceutical Profiling Platform (UDOPP), Uppsala University, BMC, Box 580, SE-75123 Uppsala, Sweden; 2 Department of Pharmacy and SciLifeLab Drug Discovery and Development Platform, ADME of Therapeutics facility, Department of Pharmacy, Uppsala University, BMC, Box 580, SE-75123 Uppsala, Sweden

## Abstract

The clinical impact of drug-drug interactions based on time-dependent inhibition of cytochrome P450 (CYP) 3A4 has often been overpredicted, likely due to use of improper inhibitor concentration estimates at the enzyme. Here, we investigated if use of cytosolic unbound inhibitor concentrations could improve predictions of time-dependent drug-drug interactions. First, we assessed the inhibitory effects of ten time-dependent CYP3A inhibitors on midazolam 1′-hydroxylation in human liver microsomes. Then, using a novel method, we determined the cytosolic bioavailability of the inhibitors in human hepatocytes, and used the obtained values to calculate their concentrations at the active site of the enzyme, i.e. the cytosolic unbound concentrations. Finally, we combined the data in mechanistic static predictions, by considering different combinations of inhibitor concentrations in intestine and liver, including hepatic concentrations corrected for cytosolic bioavailability. The results were then compared to clinical data. Compared to no correction, correction for cytosolic bioavailability resulted in higher accuracy and precision, generally in line with those obtained by more demanding modelling. The best predictions were obtained when the inhibition of hepatic CYP3A was based on unbound maximal inhibitor concentrations corrected for cytosolic bioavailability. Our findings suggest that cytosolic unbound inhibitor concentrations improves predictions of time-dependent drug-drug interactions for CYP3A.

## Introduction

Harmful drug-drug interactions (DDIs) are often caused by inhibition of cytochrome P450 (P450) enzymes in hepatocytes. Inhibition of these enzymes depends on roughly two types of mechanisms: reversible (direct) and irreversible (time-dependent). Because time-dependent inhibition can manifest itself in a slow onset and a long-lasting inhibition *in vivo*^1^, a correct assessment of *in vitro* inhibition values and an accurate prediction of the clinical consequences are important. Static predictions of time-dependent inhibition of P450 enzymes have frequently been associated with overestimations^2,3^. In recent years, however, improved system data (e.g. P450 turnover) and experimental settings have led to better confidence in time-dependent inhibition predictions for some compounds^4^. Another explanation for the overestimations associated with time-dependent inhibition of P450 enzymes has been suggested to be conservative inhibitor concentration estimates^3^. Here, we investigate if the use of cytosolic unbound drug concentrations can be used to improve the predictions further.

**Figure 1:**
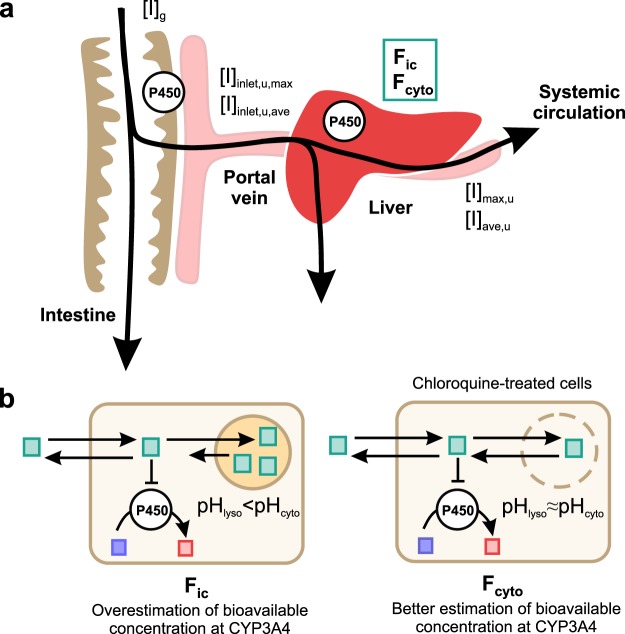
Schematic drawing of the models and the inhibitor concentrations used in the DDI predictions. (**a**) Time-dependent inhibitors can alter the activity of CYP3A in both intestine and liver. Measured or estimated total and unbound inhibitor concentrations in blood are commonly used as surrogates for the inhibitor concentrations affecting CYP3A inside cells. More realistic estimates of the intracellular unbound concentrations affecting CYP3A are obtained by correcting these concentrations for intracellular (F_ic_) or cytosolic bioavailability (F_cyto_). (**b**) Difference between F_ic_ and F_cyto_. CYP3A is located in the endoplasmic reticulum with its binding sites facing the cytosol. As the pH inside lysosomes is below that of the cytosol, basic lipophilic compounds tend to get trapped in the lysosomes. Since F_ic_ also includes drugs in the acidic endo-lysosomal compartment, F_ic_ (left) gives an overestimation of the basic lipophilic inhibitor compound available at the active site of CYP3A. F_cyto_ (right), is determined in the presence of chloroquine, which increases the lysosomal pH and eliminates the trapping of bases inside acidic compartments. As drugs access CYP3A from the cytosol, correction for F_cyto_ gives a better estimate than F_ic_ of the actual unbound drug concentration binding to the enzyme. F_cyto_, cytosolic bioavailability; F_ic_, intracellular bioavailability; [I]_ave,u_, unbound average inhibitor concentration; [I]_g_, intestinal inhibitor concentration; [I]_inlet,ave,u_, unbound hepatic inlet inhibitor concentration based on [I]_ave_; [I]_inlet,max,u_, unbound hepatic inlet inhibitor concentration based on [I]_max_; [I]_max,u_, unbound peak inhibitor concentration; P450, cytochrome P450.

Basic static prediction models are usually used to initially screen the risk of inhibition for a worst-case constant exposure of the inhibitor, e.g., its total or unbound maximal systemic concentration at steady state ([I]_max_ or [I]_max,u_)^5,6^. Mechanistic static models, which incorporate several interaction mechanisms, provide more quantitative predictions. However, no consensus has been reached on which inhibitor concentrations to use in these predictions, and several alternatives are available. These include total or unbound [I]_max_, time-averaged ([I]_ave_), or calculated liver-specific ([I]_inlet_) concentrations (Fig. 1a)^3,5,6^. In addition, although arbitrary, different concentrations are sometimes used for different interaction mechanisms (mixed term approach)^2,3^, i.e., in the same prediction, one concentration is used for reversible inhibition and another for time-dependent inhibition. Nevertheless, all these static approaches are based on systemic concentrations, which function as rough surrogates for the actual concentrations available for binding to the active site of P450 enzymes – the cytosolic unbound concentrations. Recently, a simple, robust, and label-free method was developed in our lab for determination of the intracellular bioavailability (F_ic_) of drugs^7^. F_ic_ expresses the ratio between the intracellular unbound drug concentration and the extracellular drug concentration (Fig. 1b)^8^. Our methodology, which is based on parallel intracellular accumulation and binding experiments, has been successfully used to determine drug access to intracellular targets in multiple cell systems (including primary human cells)^8,9^. F_ic_ provides a measurement of the net impact of all cellular drug disposition processes on intracellular bioavailable drug levels^8^. It correlates well with target engagement^10,11^ and pharmacological response in several therapeutic areas including cancer, inflammation and dementia^9,12^.

The use of F_ic_-adjusted drug concentrations, i.e. intracellular unbound concentrations, in DDI predictions based on time-dependent inhibition of P450 enzymes has not yet been evaluated. CYP3A4, the most important drug-metabolizing enzyme, is primarily expressed in liver and intestinal cells^13^. More specifically, it is located on the outer membrane of the endoplasmic reticulum, facing the cytosol^14–16^. A limitation of the F_ic_ method is that it provides an average estimate of intracellular bioavailability and no information on the subcellular drug distribution. For instance, positively charged compounds with lipophilic moieties tend to accumulate in lysosomes and acidic subcellular compartments where they are inaccessible to cytosolic targets (Fig. 1b)^17^. Hence, the F_ic_ of such compounds becomes inflated, resulting in an overestimation of the cytosolic bioavailability^7^. Determination of the intracellular bioavailability in the presence of chloroquine or other compounds that increase the lysosomal pH and eliminate the trapping of basic drugs inside endo-lysosomal compartments^12,18,19^ could give a better estimate for the relevant concentration at the CYP3A4 enzyme metabolic site, since it should better reflect the cytosolic bioavailability (F_cyto_).

The aim of this work was to evaluate the impact of hepatic intracellular unbound (F_ic_-corrected) and cytosolic unbound (F_cyto_-corrected) concentrations of time-dependent CYP3A inhibitors in DDI predictions. First, we determined the *in vitro* reversible and time-dependent microsomal inhibition properties of ten time-dependent CYP3A inhibitors with clinically known DDIs. Then, we determined the F_ic_ and F_cyto_ of the inhibitors in human hepatocytes. Finally, we carried out interaction predictions based on the inhibition data and a range of different inhibitor concentration combinations, which were either uncorrected or corrected for F_ic_ or F_cyto_, and compared the results to interaction data from clinical studies. We show that F_cyto_-correction of hepatic inhibitor concentrations results in predictions of the highest accuracy and precision, followed by F_ic_-corrected concentrations, and lastly uncorrected concentrations. Our data suggest that use of cytosolic and intracellular unbound inhibitor concentrations improves predictions of time-dependent DDIs.

## Results

### Drug-metabolizing enzyme inhibition

To characterize the inhibitory effects of the selected drugs on the CYP3A-mediated 1′-hydroxylation of midazolam in human liver microsomes (HLM), we first measured reversible inhibition, i.e., the inhibitory concentration causing 50% inhibition (IC_50_) of each drug, and corrected it for the measured non-specific binding to HLM. Reversible IC_50_ values ranged from 1.31 µM for the most potent inhibitor (nilotinib), to >100 µM for the weakest ones (azithromycin and crizotinib) (Table 1; Supplementary Figs S1–S2).

**Table 1:** Inhibition of CYP3A in HLM by the inhibitor compounds tested.

Compound	f_u,mic_ at 0.1 mg/ml	f_u,mic_ at 0.5 mg/ml	IC_50_ (µM)	K_i,u_^a^ (µM)	K_I_ (µM)	K_I,u_^b^ (µM)	k_inact_ (1/min)
***Strong inhibitors***							
Nefazodone	0.187	0.180	2.59 ± 0.22	0.242	4.80 ± 1.25	0.863	0.103 ± 0.013
Telithromycin	0.962	0.657	14.1 ± 3.5	6.77	1.49 ± 0.190	0.979	0.021 ± 0.001
***Moderate inhibitors***							
Crizotinib	0.505	0.118	>100	97.4	0.775 ± 0.130	0.091	0.054 ± 0.03
Diltiazem	0.792	0.708	43.9 ± 4.6	17.4	3.80 ± 1.17	2.69	0.020 ± 0.002
Erythromycin	0.640	0.586	7.78 ± 1.47	4.98	12.0 ± 1.17	7.01	0.038 ± 0.001
Imatinib	0.887	0.544	12.5 ± 2.8	5.56	3.07 ± 0.710	1.67	0.020 ± 0.001
Nilotinib	0.195	0.121	1.31 ± 0.30	0.128	1.29 ± 0.230	0.156	0.031 ± 0.001
Verapamil	0.799	0.621	2.78 ± 0.55	1.11	1.51 ± 0.460	0.937	0.042 ± 0.003
***Weak inhibitors***							
Azithromycin	0.766	0.765	>200^c^	n/a	n/a^c^	n/a	n/a^c^
Pazopanib	0.526	0.187	42.3 ± 18.7	11.1	1.88 ± 0.290	0.352	0.016 ± 0.001
Roxithromycin	0.637	0.612	82.4 ± 44.9	26.2	31.4 ± 16.5	19.2	0.021 ± 0.005

Reversible and time-dependent inhibition parameters are given together with f_u,mic_ values. The K_i,u_ and K_I,u_ values have been corrected for non-specific binding to microsomal proteins (f_u,mic_), as described in Methods. The inhibitors are classified according to their inhibitory effects on CYP3A substrates *in vivo* (see Supplementary Table S1 for references).

f_u,mic_, unbound drug fraction in the microsomal mixture; IC_50_, half maximal inhibitory concentration; K_i_, reversible inhibition constant; K_I_, inactivation rate constant; k_inact_, maximal inactivation rate. The values are given as mean ± standard deviation of triplicate measurements.

^a^Calculated as (f_u,mic_ at 0.1 mg/ml × IC_50_)/2^53^.

^b^Calculated as f_u,mic_ at 0.5 mg/ml × K_I_.

^c^At the highest concentration tested (200 µM), azithromycin caused only a 32% reversible inhibition. It did not exhibit time-dependent inhibition in K_I_/k_inact_ experiments.

**Figure 2:**
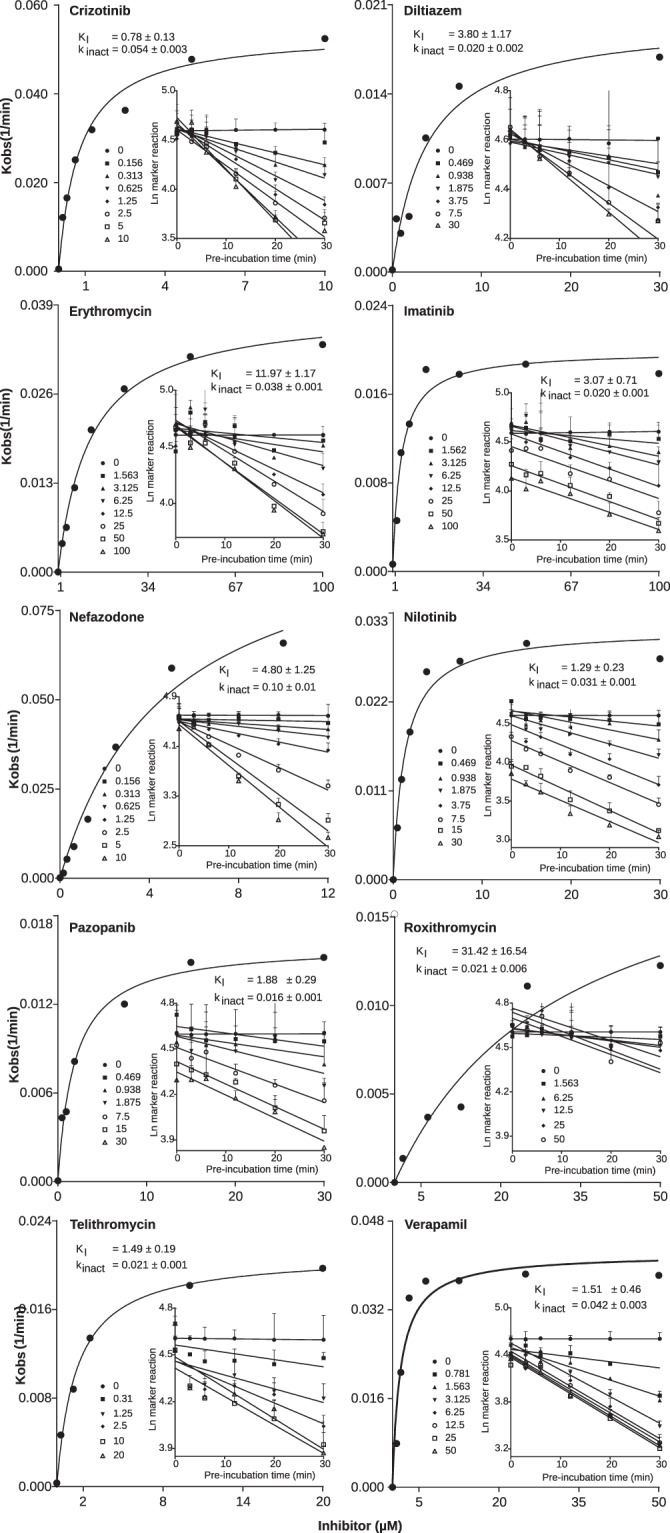
Time-dependent inhibition of CYP3A by the ten tested inhibitors in human liver microsomes (HLM). K_I_ and k_inact_ values of the inhibitors (crizotinib, diltiazem, erythromycin, imatinib, nefazodone, nilotinib, pazopanib, roxithromycin, telithromycin, verapamil) were determined in pooled HLM with midazolam 1′-hydroxylation as the marker reaction for CYP3A activity. The rate of inactivation of CYP3A activity by each inhibitor concentration (k_obs_) was determined by linear regression analysis of the natural logarithm of the percentage of activity remaining versus preincubation time data for each drug (inserts). The K_I_ and k_inact_ were then calculated by non-linear regression analysis of the k_obs_ versus inhibitor concentration. The incubations were carried out in triplicate, and the results are given as mean ± standard deviation. K_I_, inactivation rate constant; k_inact_, maximal inactivation rate; K_obs_, initial inactivation rate.

Then, we determined time-dependent inhibitory effects on CYP3A for the compounds (the maximal inactivation rate (k_inact_) and concentration resulting in half of k_inact_ (K_I_)). After correction for measured non-specific binding to HLM, the calculated K_I,u_ ranged from 0.09 to 19.2 µM, and k_inact_ from 0.016 to 0.103 1/min, which is accordance with previously published data (Table 1; Figs 2 and S2). Crizotinib had the lowest K_I,u_ value (0.09 µM), while nefazodone exhibited the highest inactivation rate (0.103 1/min). Unexpectedly, azithromycin, which has previously been classified as a time-dependent inhibitor of CYP3A^20^, did not exhibit time-dependent inhibition (data not shown). It was therefore excluded from further experiments and DDI predictions.

### Intracellular and cytosolic bioavailability (F_ic_ and F_cyto_)

To predict the inhibitor concentrations available to bind CYP3A, we determined their F_ic_ and F_cyto_ values. F_cyto_ was determined similar to F_ic_, but the intracellular accumulation (Kp) experiment was carried out in the presence of chloroquine (Kp_cyto_). Chloroquine increases lysosomal pH, and thereby reduces the trapping of lipophilic bases in this compartment^12,17^. We reasoned that this would allow a more accurate estimation of the unbound concentration in the cytoplasm.

**Figure 3:**
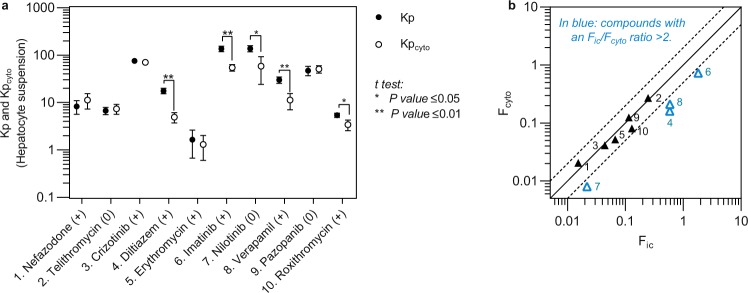
Intracellular drug accumulation in the absence (Kp) and presence (Kp_cyto_) of chloroquine (**a**) and intracellular drug bioavailability (F_ic_) and cytosolic bioavailability (F_cyto_) (**b**) of the tested inhibitors in hepatocytes. The signs within brackets in (**a**) denote charge at physiological pH. Lipophilic cationic drugs showed higher Kp than Kp_cyto_. The neutral drugs telithromycin and pazopanib had comparable Kp and Kp_cyto_, while nilotinib displayed higher Kp than Kp_cyto_, likely due to precipitation and accumulation in the lysosomes^26^. The numbers in (**b**) refer to the inhibitors listed in (**a**). Results are given as geometrical means (**a**,**b**) ± standard deviation (**a**) of triplicate measurements.

The F_cyto_ values ranged from 0.010 (nilotinib) to 0.73 (imatinib) or 73-fold (Fig. 3, Table 2). Kp was significantly larger than Kp_cyto_ (p ≤ 0.05 in Student’s t-test) for diltiazem, imatinib, nilotinib, verapamil and roxithromycin. F_cyto_ differed by more than two-fold to F_ic_ for diltiazem, imatinib, nilotinib, and verapamil. In general, F_ic_ values were low, in line with previous data in metabolically competent hepatocyte suspensions^8^.

**Table 2:** Physicochemical characteristics and intracellular drug bioavailability (F_ic_) and cytosolic bioavailability (F_cyto_) of the inhibitors.

Drug	MW (g/mol)^a^	Basic pKa^a^	Net charge at pH 7.4^a^	Log P^a^	Log D_(pH 7.4)_^a^	PSA (Å^2^)^a^	f_u,cell_	Kp	Kp_cyto_	F_ic_	F_cyto_	F_ic_/F_cyto_
Nefazodone	470.0	7.35	+	4.44	4.17	55.5	0.0019 ± 0.0001	8.1 ± 1.3	11 ± 1.6	0.015 ± 0.002	0.021 ± 0.003	0.74
Telithromycin	812.0	8.63 (b); 1.63 (a)	0	3.23	3.2	172	0.0371 ± 0.0016	6.6 ± 0.6	7.24 ± 0.8	0.25 ± 0.03	0.27 ± 0.03	0.92
Crizotinib	450.4	9.65	+	4.23	1.99	78	0.0006 ± 0.0001	75 ± 0.11	70 ± 0.4	0.044 ± 0.005	0.041 ± 0.005	1.07
Diltiazem	414.5	8.33	+	3.65	2.67	59.1	0.0335 ± 0.0024	17.48 ± 0.8	4.79 ± 0.8	0.585 ± 0.050	0.161 ± 0.030	3.65
Erythromycin	733.9	8.63	+	2.3	1.07	194	0.0444 ± 0.0013	1.5 ± 2.4	1.2 ± 4.33	0.066 ± 0.104	0.052 ± 0.192	1.28
Imatinib	493.6	8.20	+	4.15	3.29	86.3	0.0135 ± 0.0001	135 ± 3	54 ± 2.2	1.82 ± 0.05	0.73 ± 0.03	2.50
Nilotinib	529.5	5.45	0	5.00	5.00	97.6	0.0002 ± 0.0000	136 ± 4	51 ± 9	0.022 ± 0.001	0.008 ± 0.001	2.70
Verapamil	454.6	8.46	+	4.45	3.35	64	0.0198 ± 0.0010	30 ± 1.4	11 ± 2.0	0.59 ± 0.04	0.21 ± 0.04	2.78
Pazopanib	437.5	5.14	0	3.46	3.46	119	0.0025 ± 0.0000	46 ± 3	50 ± 2.2	0.11 ± 0.01	0.12 ± 0.01	0.92
Roxithromycin	837.1	8.40	+	3.17	2.13	217	0.0241 ± 0.0004	5.4 ± 0.3	3.3 ± 0.75	0.13 ± 0.01	0.08 ± 0.02	1.61

F_ic_ values were calculated as the product of intracellular fraction of unbound drug (f_u,cell_) and cellular drug accumulation ratio (Kp), which had been determined in cryopreserved human hepatocytes. Kp_cyto_ describes the cellular drug accumulation ratio of the inhibitor in the presence of chloroquine. F_cyto_ is the product of f_u,cell_ and Kp_cyto_.

F_ic_, intracellular bioavailability; F_cyto_, cytosolic bioavailability; f_u,cell_, intracellular fraction of unbound drug; Kp, cellular drug accumulation ratio; MW, molecular weight; PSA, polar surface area. Results are given as geometrical mean ± standard deviation of triplicate measurements.

^a^Obtained from ADMET Predictor (Simulations Plus, Lanchester, CA).

### Interaction predictions

**Figure 4:**
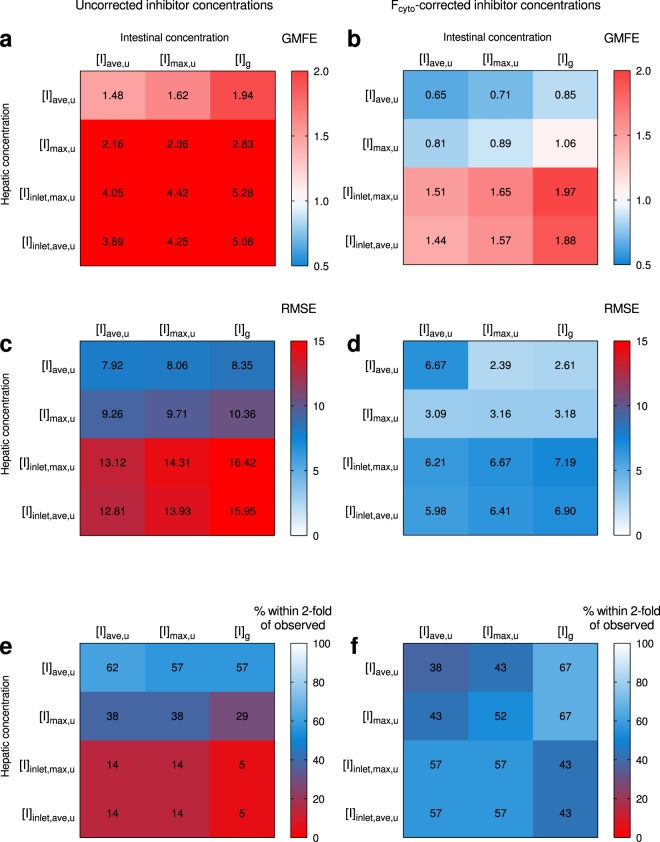
Comparison of the effects of twelve inhibitor concentration combinations on the predictions of increase in the AUCs in clinical DDI studies. Reversible inhibition and time-dependent inhibition of CYP3A were predicted using uncorrected intestinal inhibitor concentrations, and uncorrected (left panel) or F_cyto_-corrected (right panel) hepatic inhibitor concentrations. Twelve intestinal and hepatic inhibitor concentration combinations were evaluated for prediction bias (GMFE) (**a**,**b**), precision (RMSE) (**c**,**d**), and for the percentage of the predictions that were within two-fold of the observed AUC values (**e**,**f**). A perfect prediction of all actual values would give a GMFE value of 1, and a lower RMSE value denotes a greater precision of the prediction. Input parameters are given in Methods and in Supplementary Table S5. See Supplementary Table S2 for more details and results of predictions based on the mixed term approach. GMFE, geometric mean-fold error; F_cyto_, cytosolic bioavailability; [I]_ave,u_, unbound average inhibitor concentration; [I]_g_, intestinal inhibitor concentration; [I]_inlet,ave,u_, unbound hepatic inlet inhibitor concentration based on [I]_ave_; [I]_inlet,max,u_, unbound hepatic inlet inhibitor concentration based on [I]_max_; [I]_max,u_, unbound peak inhibitor concentration; RMSE, root-mean square error.

The reversible and time-dependent inhibition values for each inhibitor were combined with hepatic and intestinal inhibitor concentrations for the DDI predictions. In total, 12 hepatic and intestinal inhibitor concentration combinations were used to predict the change in AUCs of the victim drugs in 21 clinically documented interaction studies (Fig. 4, Supplementary Table S1). The different hepatic inhibitor concentrations used in the mechanistic static model (see Equation 6) were [I]_max,u_, [I]_ave,u_, [I]_inlet,max,u_, and [I]_inlet,ave,u_, respectively. The hepatic inhibitor concentrations were either used as they were (Fig. 4, left panels), or corrected for F_cyto_ (Fig. 4, right panels). The intestinal inhibitor concentrations used in the modelling were [I]_g_, [I]_max,u_, and [I]_ave,u_, respectively. The intestinal inhibitor concentrations were left uncorrected as no F_cyto_ was determined in enterocytes in this study. In addition, the effects of F_ic_-correction of hepatic inhibitor concentrations were evaluated (Supplementary Table S2).

**Figure 5:**
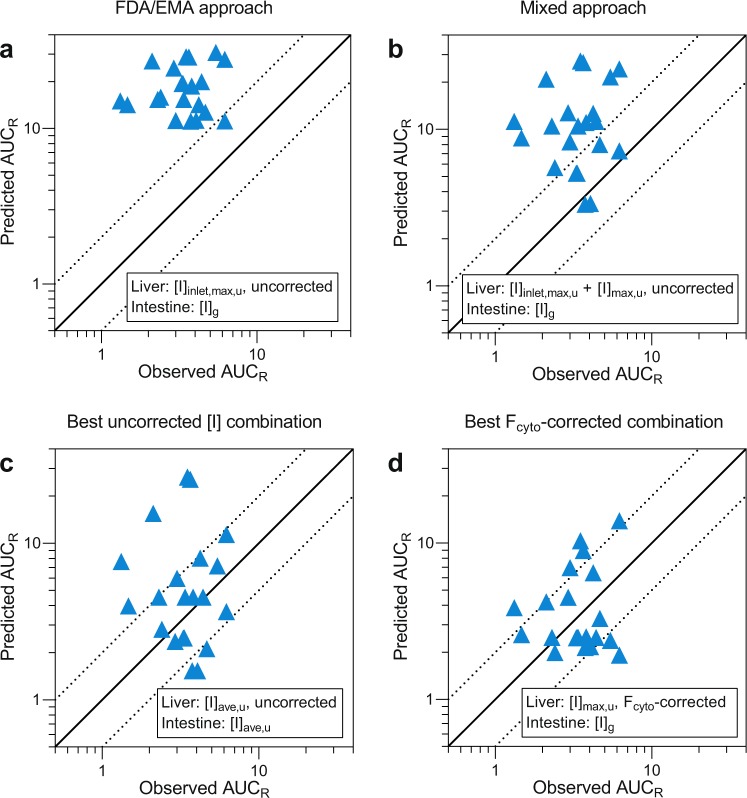
Effect of F_cyto_-correction of hepatic inhibitor concentrations on prediction accuracy. The inhibitor concentration combination in (**a**) is the one recommended in drug authority guidelines, ([I]_inlet,max,u_ + [I]_g_). The concentration combination in (**b**) corresponds to a mixed term approach, ([I]_inlet,max,u_ for reversible inhibition in the liver, [I]_max,u_ for time-dependent inhibition in the liver, and [I]_g_ for intestinal inhibition). Our data suggest that if no F_cyto_ values are available, [I]_ave,u_ for inhibition of both hepatic and intestinal CYP3A could be used (**c**). In the present study, the best prediction (GMFE closest to 1) was obtained with F_cyto_-corrected [I]_max,u_ in combination with [I]_g_ (**d**). The full line denotes no difference between predicted and observed interactions and the dashed lines indicate a prediction within two-fold of the observed values. Input parameters are given in Methods and in Supplementary Table S5. See Supplementary Table S2 for detailed results of the predictions, and Supplementary Fig. S4 for an identical figure with references to the corresponding clinical trials. AUC_R_, the area-under-the-concentration-time curve of the substrate in the presence vs absence of the inhibitor; F_cyto_, cytosolic bioavailability; [I]_ave,u_, unbound, average inhibitor concentration; [I]_g_, intestinal inhibitor concentration; [I]_inlet,max,u_, unbound hepatic inlet inhibitor concentration based on [I]_max_; [I]_max,u_, unbound, peak inhibitor concentration.

Next, the combination of inhibitory concentrations recommended by the EMA and FDA ([I]_inlet,max,u_ in combination with [I]_g_), were used as driving concentrations in the mechanistic static modelling of the AUCs obtained in the clinical interaction studies. This resulted in geometric mean fold error (GMFE; accuracy measurement) and root mean square error (RMSE; precision measurement) values of 5.28 and 16.4, respectively, and only 5% (1/21) of the interaction predictions were within 2-fold of the observed values (Figs 4–5, Supplementary Table S2). The non-physiological, mixed-term concentration combination (see above) gave slightly lower GMFE and RMSE values of 3.05 and 11.2. Twenty-nine percent (6/21) of the predictions were within 2-fold of the clinical interaction values (Fig. 5, Supplementary Table S2)^2,3^.

F_cyto_ corrections resulted in more accurate predictions of the changes in AUC and reduced the GMFE and RMSE values (Fig. 4, Supplementary Table S2). A combination of F_cyto_-corrected [I]_max,u_ for hepatic inhibition and [I]_g_ for intestinal inhibition resulted in the GMFE value closest to 1 (1.06), while the RMSE was 3.18. Further, the frequency of predictions within two-fold of the observed DDIs increased to 67% (14/21; Figs 4–5**)**. In addition, F_cyto_-corrected [I]_ave,u_ in combination with [I]_g_ gave good results with 14/21 interactions within two-fold of the clinical data (GMFE 0.85, RMSE 2.39) (Fig. 4, Supplementary Table S2).

Although they were better than uncorrected predictions, F_ic_-corrections generally did not give as good predictions as F_cyto_ (Supplementary Table S2). This can be attributed to the overestimation of the bioavailable concentration of the positively charged compounds to CYP3A. However, it should be noted that predictions based on F_ic_-corrected [I]_ave,u_ for hepatic inhibition, and [I]_g_ for intestinal inhibition resulted in predictions with 14/21 interactions within two-fold of actual data, and GMFE and RMSE values of 0.97 and 2.65 (Supplementary Table S2**)**.

To summarize, application of F_cyto_-correction of hepatic inhibitor concentrations resulted in improved DDI predictions as compared to current recommendation for static modelling that are not corrected for F_cyto_.

## Discussion

We determined the cytosolic bioavailability (F_cyto_) and *in vitro* reversible and time-dependent inhibition properties of a range of known time-dependent inhibitors of CYP3A with documented clinical interactions (Tables 1–2**)**. Data from these experiments were used, together with literature data, to predict DDIs using a mechanistic static prediction model recommended by the regulatory agencies in Europe and the US^5,6^. Twelve combinations of hepatic and intestinal inhibitor concentrations, with and without F_cyto_-correction of hepatic concentrations alone, were evaluated to identify the most accurate predictions. As a general trend, F_cyto_-correction of inhibitor concentrations led to improved predictions (Figs 4 and 5). Inhibitor concentration combinations where inhibition of hepatic CYP3A was based on F_cyto_-corrected [I]_max,u_ concentrations resulted in the best predictions. Furthermore, interaction predictions based on the inhibitor concentration combination recommended by the drug regulatory agencies ([I]_inlet,max,u_ in combination with [I]_g_)^5,6^ were markedly improved by F_cyto_-correction.

Previously, we have used intracellular bioavailability (F_ic_) to calculate intracellular concentrations^9^. F_ic_ is the net result of all processes in a living cell that determine the intracellular drug concentration, including unknown uptake and efflux mechanisms^8^. Our F_ic_-methodology requires fewer experiments, cells, and data collection than previous methods that determine intracellular drug concentrations^21–23^. However, the F_ic_ method does not provide information on the subcellular distribution of drugs. In particular, compounds with log P > 2 and a basic pKa between 6.5 and 11 tend to accumulate in acidic intracellular organelles such as lysosomes^24^, which leads to a higher F_ic_. As CYP3A enzymes are located on the cytosolic side of the endoplasmic membrane^15,16^, F_ic_ leads to an overestimation of the concentration of lysosomotropic drugs available to this enzyme. Therefore, we also determined the cellular accumulation of the inhibitors in the presence of chloroquine, which increases the lysosomal pH and eliminates the trapping of a positively charged drug inside the endo-lysosomal compartments^18^, and used it to calculate F_cyto_^7,12^. Chloroquine particularly affected the intracellular accumulation of compounds with log P values of >3 and a basic pKa around 8; these compounds exhibited F_ic_/F_cyto_ ratios of more than 1.6-fold (Table 2, Figs 3 and S3). Given that, the endo-lysosomal volume in cells is only 0.5% as compared to the total cellular volume, this represents a substantial accumulation^25^. Also the bioavailability of nilotinib was markedly reduced by chloroquine, despite a relatively low pKa (pKa 5.5, log P 5). However, this is in line with previous findings showing that nilotinib accumulates in lysosomes because of its poor solubility at acidic pH, leading to precipitation inside the lysosome^26^.

While F_cyto_-corrected (cytosolic unbound) concentrations resulted in better overall predictions than F_ic_-corrected (intracellular unbound) concentrations (Supplementary Table S2), a limitation of our approach is the possible interaction of chloroquine with drug transporters and enzymes that might affect the intracellular disposition of the inhibitors (apart from that resulting from differences in the endo-lysosomal pH; Supplementary Table S3). As alternatives, other endo-lysosomal pH modulators (e.g. monensin or ammonium chloride)^19,27^, subcellular fractionation^22^, pH partition theory^7^, or electrochemical modeling^23^ can be used. Further studies are necessary to assess which approach gives the most accurate estimates of F_cyto_. Another limitation of our and all other studies predicting DDIs after oral administration is that we did not determine F_ic_ and F_cyto_ values of the inhibitors in primary enterocytes. Because the F_ic_ and F_cyto_ of a drug depends on multiple cell-specific processes, these values can differ between different cell types^8^. An attempt was made to evaluate the effects of intestinal inhibitor concentrations corrected with the present F_cyto_ values obtained from hepatocytes. Although these predictions were better than those based on uncorrected hepatic and intestinal concentrations (data not shown), the predictions where only hepatic concentrations had been F_cyto_-corrected were still superior. It should be noted that F_cyto_-correction of intestinal concentrations is complicated by 1) availability of primary enterocytes with physiologically relevant expression of CYP3A, 2) the fact that the levels of drug in the cell interior are influenced by both intestinal and blood drug concentrations.

**Figure 6:**
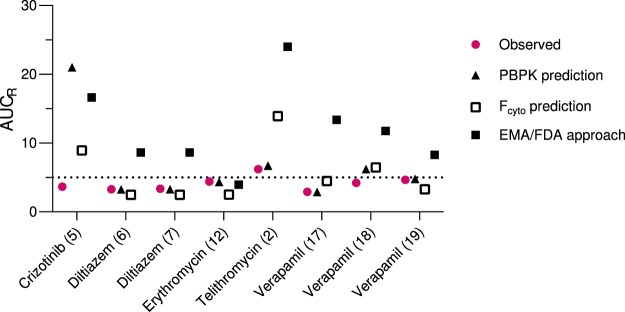
Comparison between observed clinical interaction data and predicted interaction data from PBPK simulations available in the literature and the current static model using F_cyto_-corrected hepatic concentrations ([I]_max,u_ for hepatic inhibition and [I]_g_ for intestinal inhibition) or the inhibitor concentration combination recommended by drug authorities ([I]_inlet,max,u_ + [I]_g_). The numbers within brackets on the x-axis refer to individual clinical trials listed in Supplementary Table S1. The dotted line indicates the threshold where an interaction is classified as strong (≥5-fold increase in AUC of the victim drug). References for PBPK data: crizotinib^49^; diltiazem^50,51^; erythromycin^51^; telithromycin^30^; verapamil^29,52^.

Physiologically-based pharmacokinetic (PBPK) modelling can also be used for DDI predictions, and recent drug authority guidelines allow the use of PBPK models for the assessment of DDI potential of new drugs. Although not as straightforward as static models, the strength of PBPK predictions is that they consider the dynamic changes in drug concentrations with time. However, the development of an adequate PBPK model requires time and extensive information on the pharmacokinetics and physicochemical properties of the drug. We suggest that F_cyto_ and F_ic_ values could be valuable parameters for incorporation also into PBPK models of DDIs. As F_ic_ and F_cyto_ represent the net result of all cellular drug disposition processes, single mechanisms do not have to be identified when building a new PBPK model. For instance, the Simcyp Simulator, a commonly used platform for PBPK predictions, includes an optional term called ‘active hepatic scalar’, which in its essence describes the ratio between the ‘unbound intra-hepatocyte fluid concentration of drug and the unbound blood concentration of the drug’^28^. F_cyto_ and/or F_ic_ provide exact experimental data to this parameter. Indeed, the clinical effects of several of the inhibitor compounds tested here, e.g. diltiazem, telithromycin and verapamil, have previously been predicted in PBPK simulations based on microsomal inhibition data very similar to ours (Fig. 6)^29–31^. In general, these simulations predicted the observed interactions very accurately. The exception was crizotinib, which was markedly overpredicted by microsomal data but not by hepatocyte data. Although our prediction for telithromycin resulted in a 2-fold overprediction of the observed interaction, our F_cyto_–based predictions were generally in line with those obtained by PBPK modelling. Based on this analysis, we speculate that F_cyto_ and F_ic_ could reduce the need for unnecessary parameter estimations and inclusion of undefined correction factors in PBPK models in order to obtain realistic predictions.

In the present study, no attempt was made to differentiate between inhibition of CYP3A4 or CYP3A5. The pooled microsomes used were from donors of predominantly Caucasian origin (86%), whom typically have low CYP3A5 expression levels in comparison to CYP3A4 levels, and a higher prevalence of the less ‘functional’ CYP3A5*3/*3^32,33^. Therefore, we assumed that the inhibition parameters determined were mainly reflective of CYP3A4 inhibition. However, if the role of CYP3A5 inhibition was not accurately represented or that of CYP3A4 over accounted for, this may have implications for the clinical interaction risk predictions.

Given the limited number of interactions (n = 21) evaluated, we cannot confidently propose which of the inhibitor concentration combinations that should be used in the mechanistic static DDI predictions. Nevertheless, the F_cyto_-corrected hepatic inhibitor concentrations led to improved predictions, with the combination of F_cyto_-corrected [I]_max,u_ for hepatic CYP3A inhibition and [I]_g_ for intestinal CYP3A inhibition resulting in the best prediction. Hepatic inhibitor concentrations corrected for F_ic_ also gave better predictions than those based on uncorrected concentrations only (Supplementary Table S2). Thus, our data suggest that F_cyto_ provides a good estimate of the biorelevant inhibitor concentrations in time-dependent interaction predictions.

In conclusion, we show that the overpredictions of DDIs based on time-dependent inhibition of CYP3A can be overcome by applying cytosolic unbound inhibitor concentrations in the predictions. A combination of F_cyto_ determination and inhibition experiments could thus become a powerful tool for DDI predictions. The unbound drug concentration at the site of the action is the concentration that engages the target (CYP3A), and this concentration should be used in static and dynamic prediction models and also in other pharmacokinetic models.

## Methods

### Chemicals and reagents

Crizotinib, diltiazem, erythromycin, imatinib, nilotinib, pazopanib, roxithromycin, and verapamil were obtained from Sigma-Aldrich (St. Louis, MO). Nefazodone and azithromycin were purchased from Toronto Research Chemicals (Toronto, ON) and telithromycin from Cayman Chemical Company (Ann Arbor, MI). Midazolam was obtained from Cerilliant Corporation (Round Rock, TX), 1′-hydroxymidazolam from Ultrafine Chemical Co. (Manchester, England) and nicotinamide adenine dinucleotide phosphate (NADPH) from AppliChem (Darmstadt, Germany). HLM from 50 individual mixed-sex donors were purchased from Xenotech (Lenexa, KS). Human hepatocytes were isolated *in house* from liver tissue obtained from a female donor undergoing surgical resection at the Department of Surgery, Uppsala University Hospital (Sweden) and cryopreserved as described previously^34,35^. Ethical approval was granted by the Uppsala Regional Ethics Committee (ethical Approvals Nos 2009/028 and 2011/037). Donors gave informed consent and all studies were performed in accordance with the current national regulations and ethical guidelines.

### Selection of drugs

Eleven time-dependent inhibitors of CYP3A were selected on the basis of data from the University of Washington Drug Interaction Database (http://www.druginteractioninfo.org/). These inhibitors were selected because they have documented, clinically relevant effects on the plasma concentrations of the commonly used *in vivo* CYP3A substrate drugs midazolam and simvastatin (Supplementary Table S1). The eleven compounds were: nefazodone and telithromycin (strong inhibitors of CYP3A, causing a ≥5-fold increase in the area under the concentration-time curve (AUC) of the probe drug); crizotinib, diltiazem, erythromycin, imatinib, nilotinib and verapamil (medium inhibitors causing a ≥2 but <5-fold increase); and azithromycin, pazopanib and roxithromycin (weak inhibitors causing a ≥1.25 but <2-fold increase).

### Inhibition in human liver microsomes

Given that all time-dependent inhibitors also affect the enzyme by reversible inhibition, the reversible IC_50_ value of each inhibitor was determined. Various concentrations of the inhibitors (or solvent control) were premixed with midazolam at its Michaelis-Menten concentration (K_m_; 2 µM) and HLM (0.1 mg/ml) in potassium phosphate buffer (0.1 M, pH 7.4) for 3 min (37 °C). Following inclusion of NADPH (1 mM), which initiated the reactions, the mixtures were further incubated for 5 min. Finally, the reactions were stopped by adding ice-cold acetonitrile (150 µl) containing internal standard (warfarin) to the incubation wells. The plates were put on ice for at least 10 min before further sample handling and analysis.

Time-dependent inhibition was assessed by first pre-incubating various inhibitor concentrations (or solvent control) with HLM (0.5 mg/ml) and NADPH (1 mM) in 100 mM potassium phosphate buffer, pH 7.4 for 0, 3, 6, 12, 20 and 30 min. At the determined time points, aliquots of the mixtures were transferred to separate wells containing a high concentration of midazolam (20 µM; to minimize reversible inhibition) and NADPH (1 mM), diluting the pre-incubation mixtures 20-fold. The resulting mixtures were further incubated for 5 min before addition of ice-cold acetonitrile containing internal standard (warfarin), to terminate the reactions.

Then, the unbound fraction of the inhibitors in microsomes (f_u,mic_) was determined as described below.

### Non-specific binding of drugs in human liver microsomes

The unbound fraction of the inhibitors in the microsomes (f_u,mic_) was determined using two-chambered Rapid Equilibrium Dialysis (RED) devices (Thermo Fisher Scientific Inc., Rockford, IL). Triplicate mixtures (200 µl) of 6–8 inhibitors at a final concentration of 1 µM and HLM (0.1 or 0.5 mg/ml) were added to one chamber, and Hank’s buffered salt solution (HBSS) (350 µl) to the other chamber. The dialysis units were incubated at 37 °C for 4 h on an orbital shaker. At the end of the incubation, 20 μl of each sample from the homogenate and buffer chambers was transferred to a 96-well plate. Blank buffer or blank microsomes (20 μl) were added to the samples from the homogenate or buffer chambers, respectively, to yield identical matrices before further sample handling and analysis. The f_u,mic_ values were calculated as the ratio of the drug concentration in the buffer chamber (containing drug that has passed through the dialysis membrane) to the drug concentration in the microsome chamber. To ensure that no drug had been metabolized/hydrolyzed during incubation, a stability test was performed. Here, drug-HLM mixture (100 µl) was transferred to an empty well of the dialysis unit and incubated for the same time. The remaining HLM solution (without drug) was kept as a blank sample. The mass balances calculated at the end of the experiment were consistently >80%.

### Intracellular and cytosolic drug bioavailability

Intracellular drug binding (f_u,cell_, fraction of unbound drug inside cells) and intracellular drug accumulation (Kp) were measured in two separate experiments for all inhibitors^8^, as described in the following sections. Intracellular drug bioavailability (F_ic_) expresses the ratio between the intracellular unbound drug concentration (C_u,cell_) to the drug concentration in the medium (C_medium_). F_ic_ can be calculated as the product of f_u,cell_ and Kp^9^:

1 $${{\rm{F}}}_{{\rm{ic}}}=\frac{{{\rm{C}}}_{{\rm{u}},{\rm{cell}}}}{{{\rm{C}}}_{{\rm{medium}}}}=\frac{{{\rm{f}}}_{{\rm{u}},{\rm{cell}}}\times {{\rm{C}}}_{{\rm{cell}}}}{{{\rm{C}}}_{{\rm{medium}}}}={{\rm{f}}}_{{\rm{u}},{\rm{cell}}}\,.{\rm{Kp}}$$

If incubations for determining Kp are performed in buffer, F_ic_ is equivalent to the unbound drug accumulation ratio (Kp_uu_), assuming that binding in the medium is negligible^7,36^. F_cyto_ was calculated similarly as F_ic_ (Equation 1), but the Kp value used was the one determined in the presence of chloroquine (Kp_cyto_):

2 $${{\rm{F}}}_{{\rm{c}}{\rm{y}}{\rm{t}}{\rm{o}}}={f}_{{\rm{u}},{\rm{c}}{\rm{e}}{\rm{l}}{\rm{l}}}.{{\rm{K}}{\rm{p}}}_{cyto}$$

#### Determination of intracellular fraction of unbound drug (f_u,cell_)

Cryopreserved human hepatocytes were thawed at 37 °C and transferred to phosphate-buffered saline (PBS). The cells were centrifuged for 5 min at 100 × *g* and the pellet was suspended to 10 × 10^6^ cell/ml in PBS (viability 85%) and homogenized for 10 seconds with a VCX-500 ultrasonic processor (Sonica & Materials. Newton, CT) at 20% intensity. The f_u,cell_ values were measured using two-chambered Rapid Equilibrium Dialysis (RED) devices. Cell homogenate (200 µl) containing 6–7 of the tested inhibitors, chosen at random, was transferred to one chamber and 350 µl HBSS to the other, before incubation at 37 °C for 4 h on an orbital shaker^37^. At the end of the incubation, 20 μl of each sample from the homogenate and buffer chambers was transferred to a 96-well plate. Blank buffer or blank homogenate (20 μl) was added to the samples from the homogenate or buffer chambers, respectively, to yield identical matrices. For the stability test, the cell homogenate and drug mixture (100 µl) was transferred to an empty well of the dialysis unit. The mass balances were generally >80%. After incubation, 10-fold and 100-fold dilutions of cell homogenate were prepared on 96-well plates and a mixture of acetonitrile/water (60:40) with internal standard (warfarin) was added to the samples before further sample handling and analysis, which was performed as described above. The f_u,hom_ values were calculated as the ratio of the concentration of the drug in the HBSS chamber (C_buffer_) vs. the cell homogenate chamber (C_hom_).

The protein concentration in the cell homogenate was measured using a BCA Protein Assay Reagent Kit (Pierce Biotechnology, Rockford, IL), according to the manufacturer’s instruction. The total cell volume in each well for 10 × 10^6^ primary human hepatocytes was estimated from the protein concentration. As one mg of protein in primary human hepatocytes approximates to a volume of 6.5 µl^8^, the dilution factor, *D*, was calculated according to *D* = 1**/**(mg of protein × 6.5 µl/mg). Finally, f_u,cell_ values were calculated according to Equation 3:

3 $${{\rm{f}}}_{{\rm{u}},{\rm{cell}}}=\frac{1}{D\,.(1/{{\rm{f}}}_{{\rm{u}},{\rm{\hom }}}-1)+1}$$

#### Determination of intracellular drug accumulation ratio (Kp) and cytosolic drug accumulation ratio (Kp_cyto_)

Cryopreserved human hepatocytes were thawed as described above and suspended to 1 × 10^6^ cell/ml in HBSS (viability 85%). Cell suspension (100 µl) was transferred to 96-well plates (100,000 cell/well), and drug diluted in HBSS was added to give a final drug concentration of 0.5 µM. The plates were incubated on an orbital shaker (300 rpm) at 37 °C for three time points (15, 30, 45 min), to ensure sufficient time to reach steady-state. At the end of the incubation, the plates were centrifuged for 5 min at 100 × g, and medium samples (20 µl) were collected from the supernatant (C_medium_). Drug uptake was terminated by addition of ice-cold PBS followed by a washing step. Finally, the cells were lysed with acetonitrile/water (60:40) containing internal standard (warfarin) before further sample handling and analysis. The total amount of drug in the cells (A_cell_) was calculated based on the determined drug concentrations.

The protein concentration in the cell samples was measured as described above and the cell volume (V_cell_) calculated. Finally, the Kp was calculated for each drug as the ratio between the total drug concentration in the cell vs. the drug concentration in the medium (C_medium_):

4 $${\rm{Kp}}=\frac{{{\rm{A}}}_{{\rm{cell}}}/{{\rm{V}}}_{{\rm{cell}}}}{{{\rm{C}}}_{{\rm{medium}}}}$$

Kp_cyto_ was measured similarly as Kp described above; however, the incubations included chloroquine (100 µM) for 45 min to reduce the lysosomal trapping of lipophilic bases. We have previously shown that 100 µM of chloroquine is sufficient to eliminate lysosomal trapping of basic compounds^7,12^.

### Sample handling and determination of drug concentrations

The sample plates were centrifuged for 20 min at 3,500 rpm (4 °C) before analysis. The concentrations of the inhibitors and 1′-hydroxymidazolam were analyzed by ultra-performance liquid chromatography coupled to tandem mass spectrometry (UPLC-MS/MS) (Acquity, Waters Corp., USA). The compounds were separated on a BEH C18 column (2.1 × 50 mm, 1.7 µm, Waters, Ireland) at 60 °C. The mobile phases were formic acid 0.1% and acetonitrile 5% in water (mobile phase A) and formic acid 0.1% in acetonitrile (mobile phase B). The flow rate was 500 µL/min and the following gradient was used (min/%B): 0.0/5, 0.5/5, 1.2/100, 1.6/100, 2.0/5. The compounds were quantified with a triple quadrupole mass spectrometer (Xevo TQ MS, Waters Corp., USA). Transitions, cone voltages, and collision energies used are given in Supplementary Table S4.

### Data analysis

The *in vitro* incubations were carried out in triplicate. Reversible IC_50_ values were determined by non-linear regression using GraphPad Prism (version 7, Systat Software, Inc., Chicago, IL). To determine time-dependent inhibition parameters, metabolite formation in the absence of inhibitor was used as a control, and the observed rates in the presence of inhibitor were adjusted to the control for each pre-incubation time. K_I_ and k_inact_ values were estimated using nonlinear regression (Equation 5)^38,39^:

5 $${{\rm{k}}}_{{\rm{obs}}}=\frac{{{\rm{k}}}_{{\rm{inact}}}\times [{\rm{I}}]}{{{\rm{K}}}_{{\rm{I}}}+[{\rm{I}}]}$$

where k_obs_ is the initial rate of inactivation and [I] is the initial inhibitor concentration in the incubation.

### Prediction of drug-drug Interactions

Interaction predictions were carried out for the inhibitors that exhibited time-dependent inhibition of CYP3A in the *in vitro* experiments. Clinical DDI studies with the inhibitors and the CYP3A probe drugs midazolam or simvastatin (http://www.druginteractioninfo.org/) were included as reference studies (n = 21; Supplementary Table S1). For interaction predictions, all reversible IC_50_ and time-dependent K_I_ values were corrected for non-specific binding of the drugs to HLM by multiplying the value by f_u,mic_, measured at either 0.1 mg/ml HLM (IC_50_) or 0.5 mg/ml HLM (K_I_). IC_50,u_ were further divided by 2 to obtain unbound reversible inhibition constants (K_i,u_)^40^. The corrected values were assumed to be independent of drug concentration for the range used^41^.

The mechanistic static net effect model was used for interaction predictions^2,6^, to account for simultaneous reversible and time-dependent inhibition (Equation 6). The induction term has been excluded in Equation 6 as only one of the ten inhibitors tested (verapamil) has been reported to affect CYP3A by induction *in vitro* (Supplementary Table S3).

6 $${{\rm{AUC}}}_{{\rm{R}}}=(\frac{1}{[{{\rm{A}}}_{{\rm{H}}}\times {{\rm{B}}}_{{\rm{H}}}]\times {{\rm{f}}}_{{\rm{m}}}+(1-{{\rm{f}}}_{{\rm{m}}})})\times (\frac{1}{[{{\rm{A}}}_{{\rm{G}}}\times {{\rm{B}}}_{{\rm{G}}}]\times (1-{{\rm{F}}}_{{\rm{G}}})+{{\rm{F}}}_{{\rm{G}}}})$$

7 $${\rm{A}}=\frac{1}{1+\frac{[{\rm{I}}]}{{{\rm{K}}}_{{\rm{i}}}}}$$

8 $${\rm{B}}=\frac{{{\rm{k}}}_{{\rm{\deg }}}}{{{\rm{k}}}_{{\rm{\deg }}}+\frac{{{\rm{k}}}_{{\rm{inact}}}\times [{\rm{I}}]\,}{{{\rm{K}}}_{{\rm{I}}}+[{\rm{I}}]}}$$

where AUC_R_ represents the area under the plasma concentration-time curve of the affected drug in the presence and absence of the inhibitor. A and B represent reversible and time-dependent inhibition, respectively, and subscripts _H_ and _G_ denote liver and intestine, respectively^2^. The degradation rate (k_deg_) values corresponded to 0.019 1/h (half-life of 36 h) for hepatic CYP3A4 degradation and 0.030 1/h (half-life of 23 h) for intestinal CYP3A4 degradation^4^. The fraction metabolized by CYP3A4 (f_m_) and the fraction of drug escaping CYP3A4-mediated gut metabolism (F_G_) were 0.94 and 0.51, respectively, for midazolam^42,43^, and 0.90 and 0.66, respectively, for simvastatin^44,45^.

Twelve combinations of hepatic and intestinal inhibitor concentrations were tested to evaluate the effects of F_cyto_-correction on interaction predictions. As F_cyto_ describes the fraction of extracellular drug concentration that is bioavailable in the cytosol, the extracellular inhibitor concentration can be multiplied by F_cyto_ to obtain the concentration available at the enzyme. Thus, F_cyto_-correction was performed by multiplying the hepatic inhibitor concentrations with the F_cyto_ value of each inhibitor. In addition, predictions were carried out with F_ic_-corrected hepatic inhibitor concentrations. Intestinal inhibitor concentrations were left uncorrected, since we did not determine F_cyto_ (or F_ic_) in enterocytes. The hepatic inhibitor concentrations in the predictions included the maximum unbound-inhibitor concentation at steady state in blood, [I]_max,u_; the average unbound-inhibitor concentation at steady state in blood, [I]_ave,u_,; the unbound-inhibitor blood concentration in the portal vein based on [I]_max_, [I]_inlet,max,u_; and the unbound-inhibitor blood concentration in the portal vein based on [I]_ave,u_, [I]_inlet,ave,u_.

[I]_inlet,max,u_ and [I]_inlet,ave,u_ were calculated according to Equations 9 and 10 ^5,6,46^.

9 $${[{\rm{I}}]}_{{\rm{inlet}},{\rm{\max }},{\rm{u}}}={{\rm{f}}}_{{\rm{u}},{\rm{B}}}\times ({[{\rm{I}}]}_{{\rm{\max }}}+\frac{{{\rm{k}}}_{{\rm{a}}}\times \mathrm{dose}\,\times {{\rm{f}}}_{{\rm{a}}}}{{{\rm{Q}}}_{{\rm{h}}}\times {\rm{freq}}})$$

10 $${[{\rm{I}}]}_{{\rm{inlet}},{\rm{ave}},{\rm{u}}}={{\rm{f}}}_{{\rm{u}},{\rm{B}}}\times ({[{\rm{I}}]}_{{\rm{ave}}}+\frac{{{\rm{k}}}_{{\rm{a}}}\times {\rm{dose}}\times {{\rm{f}}}_{{\rm{a}}}}{{{\rm{Q}}}_{{\rm{h}}}\times {\rm{freq}}})$$

where f_u,B_ is the unbound fraction of drug in blood (calculated as f_u_ in plasma divided by the blood to plasma ratio); k_a_ represents the absorption rate constant, dose and freq denote the inhibitor dose and frequency; f_a_ is the fraction of drug absorbed; and Q_h_ is the hepatic blood flow (97 l/h)^47^.

The intestinal inhibitor concentrations included the total enterocytic concentration, [I]_g_; the maximum unbound-inhibitor concentation at steady state in blood, [I]_max,u_; and the average unbound-inhibitor concentation at steady state in blood, [I]_ave,u_. Unless available in the literature, [I]_ave,u_ was calculated according to Equation 11:

11 $${[{\rm{I}}]}_{{\rm{ave}},{\rm{u}}}={{\rm{f}}}_{{\rm{u}},{\rm{B}}}\times (\,\frac{{\rm{AUC}}}{{\rm{\tau }}})$$

where τ is the dose interval (h).

[I]_g_ was estimated using Equation 12 ^47^:

12 $${[{\rm{I}}]}_{{\rm{g}}}=\frac{{{\rm{k}}}_{{\rm{a}}}\times {\rm{d}}{\rm{o}}{\rm{s}}{\rm{e}}\,\times {{\rm{f}}}_{{\rm{a}}}}{{{\rm{Q}}}_{{\rm{g}}}\times {\rm{f}}{\rm{r}}{\rm{e}}{\rm{q}}}$$

where Q_g_ represents the blood flow in the enterocytes (18 l/h)^48^. The inhibitor-specific input parameters are given in Supplementary Table S5.

Furthermore, an inhibitor concentration combination based on the mixed term approach, i.e., using different hepatic inhibitor concentrations for reversible and time-dependent inhibition^3^ was tested.

Each individual prediction was assessed by comparing the predicted AUC_R_ to the corresponding one in the clinical reference study (predicted AUC_R_/observed AUC_R_). To assess the precision of each concentration combination, for a total of 21 individual predictions, the mean-square error (RMSE) of the predicted interactions (AUC_R_) compared to the observed AUC_R_ was calculated according to Equation 13 ^3^:

13 $${\rm{RMSE}}=\sqrt{\frac{{\sum }^{}{({\rm{Predicted}}{{\rm{AUC}}}_{{\rm{R}}}-{\rm{Observed}}{{\rm{AUC}}}_{{\rm{R}}})}^{2}}{{\rm{number}}\,{\rm{of}}\,{\rm{predictions}}}}$$

A lower RMSE value indicates a greater precision of the prediction. Moreover, to determine the accuracy of the predictions, the geometric mean fold error (GMFE) of each combination was calculated according to Equation 14^3^:

14 $${\rm{GMFE}}=10{}^{\frac{{\sum }^{}|\mathrm{log}\frac{{\rm{Predicted}}{{\rm{AUC}}}_{{\rm{R}}}}{{\rm{Observed}}{{\rm{AUC}}}_{{\rm{R}}}}|}{{\rm{number}}{\rm{of}}{\rm{predictions}}}}$$

A combination that predicts all values perfectly would thus have a GMFE value of 1; one that over- or underpredicts values on average by 2-fold would have GMFE values of 2 and 0.5, respectively.

## Supplementary information

Supplement

## Data Availability

All data generated or analyzed during this study are included in this published article or available from the corresponding author upon reasonable request.
