# Supplementary material for: Improved predictions of time-dependent drug-drug interactions by determination of cytosolic drug concentrations
Source: Sci Rep. 2019 Apr 10;9:5850. doi: 10.1038/s41598-019-42051-x (PMC6458156; doi:10.1038/s41598-019-42051-x)
Supplement: Supplementary file 1 — Supplement [file 41598_2019_42051_MOESM1_ESM.pdf]

Supporting information

Improved predictions of time-dependent drug-drug interactions by determination of cytosolic drug concentrations

Anne M. Filppula[1*], Rezvan Parvizi[1], André Mateus[1], Pawel Baranczewski[1,2] and Per Artursson[1,2]

Affiliations:

[1] Department of Pharmacy and Uppsala Drug Optimization and Pharmaceutical Profiling Platform (UDOPP), Uppsala University, BMC, Box 580, SE-75123 Uppsala, Sweden

[2] Department of Pharmacy and SciLifeLab Drug Discovery and Development Platform, ADME of Therapeutics Facility, Uppsala University, BMC, Box 580, SE-75123 Uppsala, Sweden

*Correspondence and requests for materials should be addressed to:

Anne M. Filppula
University of Helsinki
Department of Clinical Pharmacology, Biomedicum 2C
PB 20, FI-00014 HY, Helsinki, Finland,
Tel: +358 50 311 9478, email: anne.filppula@helsinki.fi

Supplementary Tables

Supplementary Table S1. *Details of the clinical trials (n=21) used as reference studies in the drug-drug interaction predictions. The inhibitors are classified into groups according to their inhibitory effects on CYP3A4 substrates observed in vivo* [1,2] .

#	Inhibitor	Dosing regimen	Victim drug	Observed AUC_R	Reference
	Strong inhibitors				
1	Nefazodone	200 mg bid (12 d)	MDZ	5.44	[3]
2	Telithromycin	800 mg qd (6 d)	MDZ	6.20	[4]
	Moderate inhibitors				
3	Crizotinib	100 mg qd (28 d)	MDZ	2.12	[5]
4	Crizotinib	300 mg bid (28 d)	MDZ	3.50	[5]
5	Crizotinib	250 mg bid (28 d)	MDZ	3.65	[5]
6	Diltiazem	240 mg qd (5 d)	MDZ	3.29	[6]
7	Diltiazem	240 mg qd (2 d)	MDZ	3.35	[6]
8	Diltiazem	60 mg tid (2 d)	MDZ	4.06	[6]
9	Diltiazem	60 mg tid (2 d)	MDZ	3.75	[7]
10	Erythromycin	200 mg qid (2 d)	MDZ	2.32	[8]
11	Erythromycin	200 mg qid (4 d)	MDZ	3.32	[8]
12	Erythromycin	500 mg tid (7 d)	MDZ	4.42	[9]
13	Erythromycin	500 mg tid (5 d)	MDZ	3.81	[10]
14	Erythromycin	500 mg tid (2 d)	Sim	6.22	[11]
15	Imatinib	400 mg qd (7 d)	Sim	2.92	[12]
16	Nilotinib	400 mg bid (12 d)	MDZ	2.40	[13]
17	Verapamil	80 mg tid (2 d)	MDZ	2.92	[7]
18	Verapamil	480 mg qd (3 d)	Sim	4.22	[14]
19	Verapamil	80 mg tid (2 d)	Sim	4.65	[11]
	Weak inhibitors				
20	Pazopanib	800 mg qd (17 d)	MDZ	1.32	[15]
21	Roxithromycin	300 mg qd (6 d)	MDZ	1.47	[16]

AUC_R, change in area under the plasma-concentration time curve; bid, twice daily; d, days; MDZ, midazolam; qd, once daily; qid, four times per day; tid, three times per day; Sim, simvastatin.

Supplementary Table S2. *Details of the inhibitor concentration combinations used in drug-drug interaction predictions and the prediction precision (RMSE) and accuracy (GMFE) as well as the number of predictions within 2-fold of observed clinical value of each combination. The effects of reversible inhibition (RI) and time-dependent inhibition (TDI) of CYP3A4 were predicted using uncorrected intestinal inhibitor concentrations, and uncorrected or* F_{cyto} *or* F_{ic} *corrected hepatic inhibitor concentrations. A lower RMSE value indicates a greater precision of the prediction. A combination that predicts all values perfectly would have a GMFE value of 1. Input parameters are given in* ***Materials and Methods*** *and in* ***Tables 1-2*** *in the main manuscript, and in* ***Supplementary Table S5****.*

#	Liver concentration	Gut concentration	RMSE (un-corrected)	RMSE (F_{cyto}-corrected)	RMSE (F_{ic}-corrected)	GMFE (un-corrected)	GMFE (F_{cyto}-corrected)	GMFE (F_{ic}-corrected)	No of predictions within 2-fold of actual (uncorrected)	No of predictions within 2-fold of actual (F_{cyto}-corrected)	No of predictions within 2-fold of actual (F_{ic}-corrected)
1	$[I]_{max,u}$	$[I]_{ave,u}$	9.26	3.09	3.38	2.16	0.81	0.97	8/21	9/21	11/21
2	$[I]_{max,u}$	$[I]_{g}$	10.36	3.18	3.70	2.83	1.06	1.26	6/21	14/21	10/21
3	$[I]_{max,u}$	$[I]_{max,u}$	9.71	3.16	3.55	2.36	0.89	1.06	7/21	11/21	10/21
4	$[I]_{inlet,max,u}$	$[I]_{ave,u}$	13.12	6.21	7.11	4.05	1.51	1.87	3/21	12/21	9/21
5	$[I]_{inlet,max,u}$	$[I]_{g}$	16.42	7.19	9.10	5.28	1.97	2.44	1/21	9/21	7/21
6	$[I]_{inlet,max,u}$	$[I]_{max,u}$	14.31	6.67	7.86	4.42	1.65	2.04	3/21	12/21	8/21
7	$[I]_{ave,u}$	$[I]_{ave,u}$	7.92	2.61	2.77	1.48	0.65	0.74	13/21	8/21	10/21
8	$[I]_{ave,u}$	$[I]_{g}$	8.35	2.39	2.65	1.94	0.85	0.97	12/21	14/21	14/21
9	$[I]_{ave,u}$	$[I]_{max,u}$	8.06	2.52	2.72	1.62	0.71	0.81	12/21	9/21	11/21
10	$[I]_{inlet,ave,u}$	$[I]_{ave,u}$	12.81	5.98	6.88	3.89	1.44	1.79	3/21	12/21	10/21
11	$[I]_{inlet,ave,u}$	$[I]_{g}$	15.95	6.90	8.79	5.08	1.88	2.34	1/21	9/21	6/21
12	$[I]_{inlet,ave,u}$	$[I]_{max,u}$	13.93	6.41	7.59	4.25	1.57	1.95	3/21	12/21	8/21

13	*Mixed approach* $[I]_{inlet,max,u}$ (RI), $[I]_{max,u}$ (TDI)	$[I]_g$	11.20	3.34	4.00	3.05	1.08	1.30	6/21	14/21	10/21

F_{cyto}, cytosolic bioavailability; F_{ic}, intracellular bioavailability GMFE, geometric mean-fold error; $[I]_{ave}$, average inhibitor concentration; $[I]_g$, intestinal inhibitor concentration; $[I]_{inlet,max}$ hepatic inlet inhibitor concentration based on peak inhibitor concentration; $[I]_{inlet,ave,u}$, hepatic inlet inhibitor concentration based on average inhibitor concentration; $[I]_{max}$, peak inhibitor concentration; RI, reversible inhibition; RMSE, root-mean square error; TDI, time-dependent inhibition; $_u$, unbound.

Supplement

Supplementary Table S3. *Metabolism and transport properties of the inhibitor compounds included in interaction predictions. The data has been compiled from the UW Metabolism and Transport Drug Interaction Database (DIDB; Copyright University of Washington 1999-2015. UW Metabolism and Transport Drug Interaction Database, accessed: February 2017). If the compound is metabolized by CYP2D6, it may affect Kp_{cyto} results as chloroquine is a CYP2D6 inhibitor ($IC_{50} \approx 50$ µM in HLM (DIDB)). Chloroquine also weakly inhibits CYP3A4 ($IC_{50} = 150$ µM). Furthermore, it inhibits several transporters, including OATP1A2 ($IC_{50} \approx 10$ µM), OCT1 (IC_{50}=13 µM), OCT2 ($IC_{50} \approx 100$ µM), MATE1 ($IC_{50} = 1.5$ µM), MATE2-K ($IC_{50} = 4.0$ µM) and P-gp ($K_i = 54$ µM) (DIDB).*

Drug	Active metabolite	Inducer of CYP3A *in vitro*	Substrate of CYP2D6 *in vitro* and *in vivo*	Substrate of CYP3A *in vitro* and *in vivo*	Substrate of P-gp *in vitro*	Substrate of other transporters *in vitro*	SMILES
Nefazodone				●			CCC1=NN(CCCN2CCN(CC2)C2=CC(Cl)=CC=C2)C(=O)N1CCOC1=CC=CC=C1
Telithromycin				●	●		[H][C@@]12[C@@H](C)C(=O)[C@H](C)C[C@@](C)(OC)[C@H](O[C@@H]3O[C@H](C)C[C@@H]([C@H]3O)N(C)C)[C@@H](C)C(=O)[C@@H](C)C(=O)O[C@H](CC)[C@@]1(C)OC(=O)N2CCCCN1C=NC(=C1)C1=CC=CN=C1
Crizotinib				●	●	OATP1B1/3, 2B1	[H][C@](C)(OC1=CC(=CN=C1N)C1=CN(N=C1)C1CCNCC1)C1=C(Cl)C=CC(F)=C1Cl
Diltiazem	●		●	●	●		COC1=CC=C(C=C1)[C@@H]1SC2=C(C=CC=C2)N(CCN(C)C)C(=O)[C@@H]1OC(C)=O
Erythromycin				●	●	BCRP, MRP2/3, OAT2, OATP1A2, 1B1/3	CC[C@H]1OC(=O)[C@H](C)[C@@H](O[C@H]2C[C@@](C)(OC)[C@@H](O)[C@H](C)O2)[C@H](C)[C@@H](O[C@@H]2O[C@H](C)C[C@@H]([C@H]2O)N(C)C)[C@](C)(O)C[C@@H](C)C(=O)[C@H](C)[C@@H](O)[C@]1(C)O
Imatinib				●	●	BCRP, MATE1, MRP4, OATP1A2, 1B3, OCT1/2	CN1CCN(CC2=CC=C(C=C2)C(=O)NC2=CC(NC3=NC=CC(=N3)C3=CN=CC=C3)=C(C)C=C2)CC1
Nilotinib				●	●	OATP1B1/3, OCT1	CC1=CN(C=N1)C1=CC(=CC(NC(=O)C2=CC(NC3=NC=CC(=N3)C3=CN=CC=C3)=C(C)C=C2)=C1)C(F)(F)F
Verapamil	●	(●)		●	●	MATE2-K	COC1=C(OC)C=C(CCN(C)CCCC(C#N)(C(C)C)C2=CC(OC)=C(OC)C=C2)C=C1
Pazopanib				●	●	OATP1B1/3, OCT1	CN(C1=CC2=NN(C)C(C)=C2C=C1)C1=CC=NC(NC2=CC=C(C)C(=C2)S(N)(=O)=O)=N1
Roxithromycin					●		CC[C@H]1OC(=O)[C@H](C)[C@@H](O[C@H]2C[C@@](C)(OC)[C@@H](O)[C@H](C)O2)

	[C@H](C)[C@@H](O[C@@H]2O[C@H](C)C[C@@H]([C@H]2O)N(C)C)[C@](C)(O)C[C@@H](C)C(=NOCOCCOC)[C@H](C)[C@@H](O)[C@]1(C)O

Supplementary Table S4. *Mass spectrometric properties for quantification of compounds.*

Compound	Parent m/z	Daughter m/z	Cone voltage	Collision energy	Ionization mode
1-OH-Midazolam	342.1	203.0	34	28	ESI+
Azithromycin	749.3	40	82.7	50	ESI+
Crizotinib	450.2	260.1	29	25	ESI+
Crizotinib	450.2	177.0	29	39	ESI+
Diltiazem	416.0	177.9	28	28	ESI+
Erythromycin	734.4	158.1	24	30	ESI+
Erythromycin	734.4	83.0	24	44	ESI+
Imatinib	494.3	394.1	42	26	ESI+
Imatinib	494.3	217.1	42	24	ESI+
Nefazodone	471.1	275.0	46	28	ESI+
Nilotinib	530.0	288.9	46	28	ESI+
Pazopanib	439.0	358.0	40	28	ESI+
Roxithromycin	838.4	158.0	28	34	ESI+
Telithromycin	813.3	115.9	40	46	ESI+
Verapamil	456.1	164.9	46	28	ESI+

Supplementary Table S5. *Inhibitor parameters in the drug-drug interaction predictions.*

Parameter	Crizotinib	Diltiazem	Erythromycin	Imatinib	Nefazodone	Nilotinib	Pazopanib	Roxithromycin	Telithromycin	Verapamil
MW (g/mol)	450.339	414.52	733.94	493.603	470.01	529.53	437.52	837.05	812.02	454.60
B/P	1.1	0.963[a]	0.854[a]	0.732	0.73[a]	0.76	0.76	0.70[a]	0.70	0.76
$f_{u,p}$	0.093	0.20	0.31	0.05	0.01	0.001	0.001	0.10	0.30	0.10
F_a	0.4	1.00	0.65	0.98	1.00	0.30	0.80	0.86	0.92	1.00[a]
k_a (1/h)	0.817	1.02[a]	0.6	1.1	6[b]	6[b]	6[b]	4.96	0.95	1.2
$C_{max,ss}$ (ng/ml)	86 (100 mg qd), 327 (250 mg bid), 420 (300 mg bid)	166 (240 mg qd), 70 (60 mg tid)	821 (500 mg tid)	2,596 (400 mg qd)	1,400 (200 mg bid)	1,595 (400 mg bid)	45,000 (800 mg qd)	10,900 (300 mg qd)	1,840 (800 mg qd)	230 (80 mg tid), 875 (240 mg bid)
$C_{ave,ss}$ (ng/ml)	45 (100 mg qd), 169 (300 mg bid), 129 (250 mg bid)	100 (240 mg qd), 37 (60 mg tid)	361 (500 mg tid)	1,671 (400 mg qd)	582 (200 mg bid)	1,138 (400 mg bid)	30,971 (800 mg qd)	5,413 (300 mg qd)	425 (800 mg qd)	33 (80 mg tid), 348 (240 mg bid)
References	[5,17]	[6,18]	[19-21]	[22-25]	[26-28]	[29,30]	[29,31]	[20,32,33]	[4,34]	[35-40]

B/P, blood-to-plasma ratio; bid, twice daily; $C_{ave,ss}$, average plasma concentration at steady-state; $C_{max,ss}$, maximum plasma concentration at steady-state; F_a, fraction absorbed; $f_{u,p}$, fraction of unbound in plasma; k_a, absorption rate constant; MW, molecular weight; qd, once daily; qid, four times daily; tid, three times daily.

[a] Predicted within/obtained from Simcyp (Simcyp Limited, UK).

[b] When no values were available in the literature, a k_a value of 6 1/h (0.1 1/min) was used in the predictions [1].

Supplementary figures

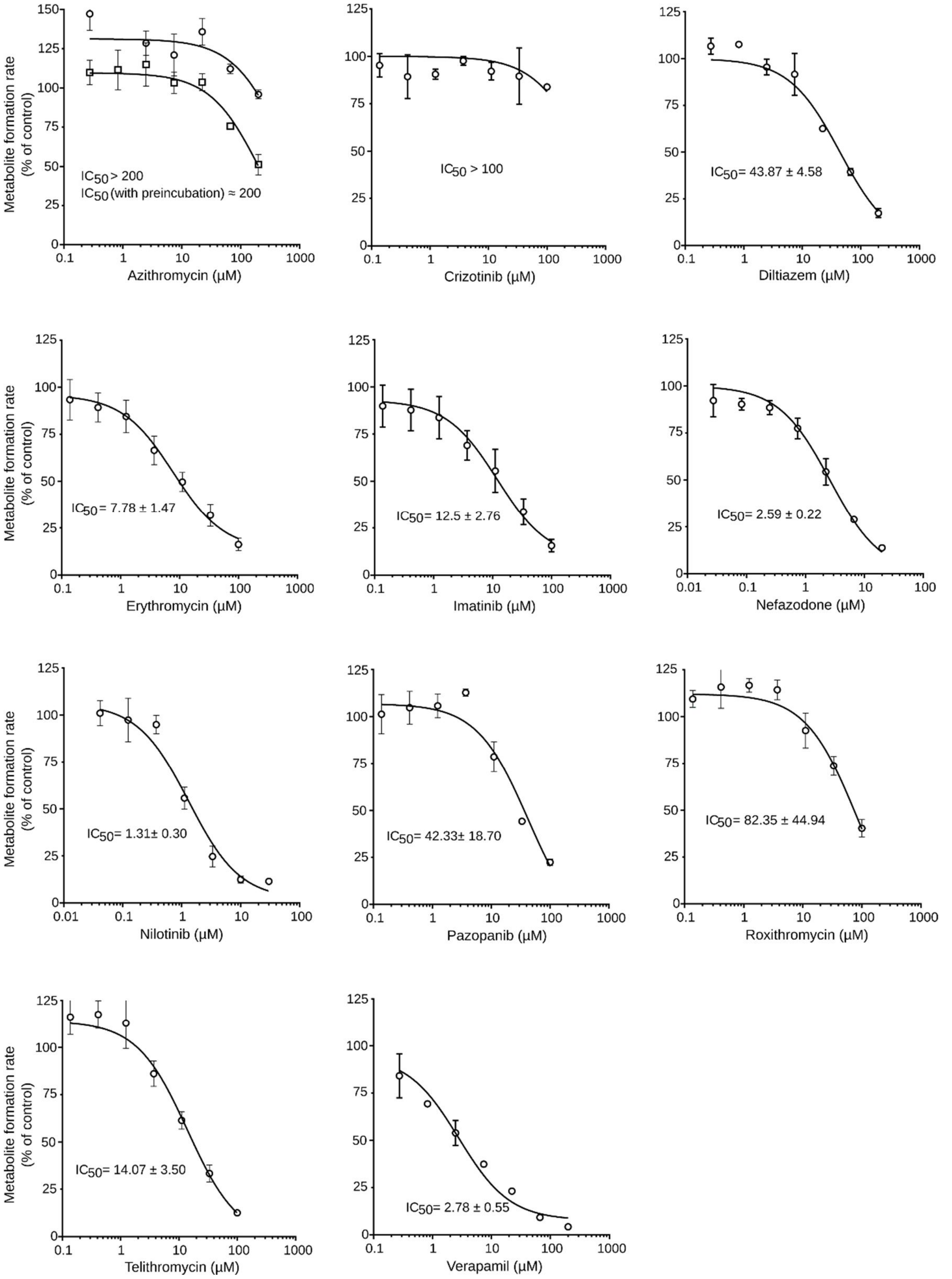

Supplementary figure S1. *The reversible inhibitory effects of the tested inhibitors on CYP3A4 in HLM. The reversible* IC_{50} *values of the inhibitors were determined in pooled HLM with midazolam 1'-hydroxylation as the marker reaction for CYP3A4 activity. The incubations were carried out as triplicates. HLM, human liver microsomes;* IC_{50}*, half maximal inhibitory concentration.* IC_{50}*, half maximal inhibitory concentration.*

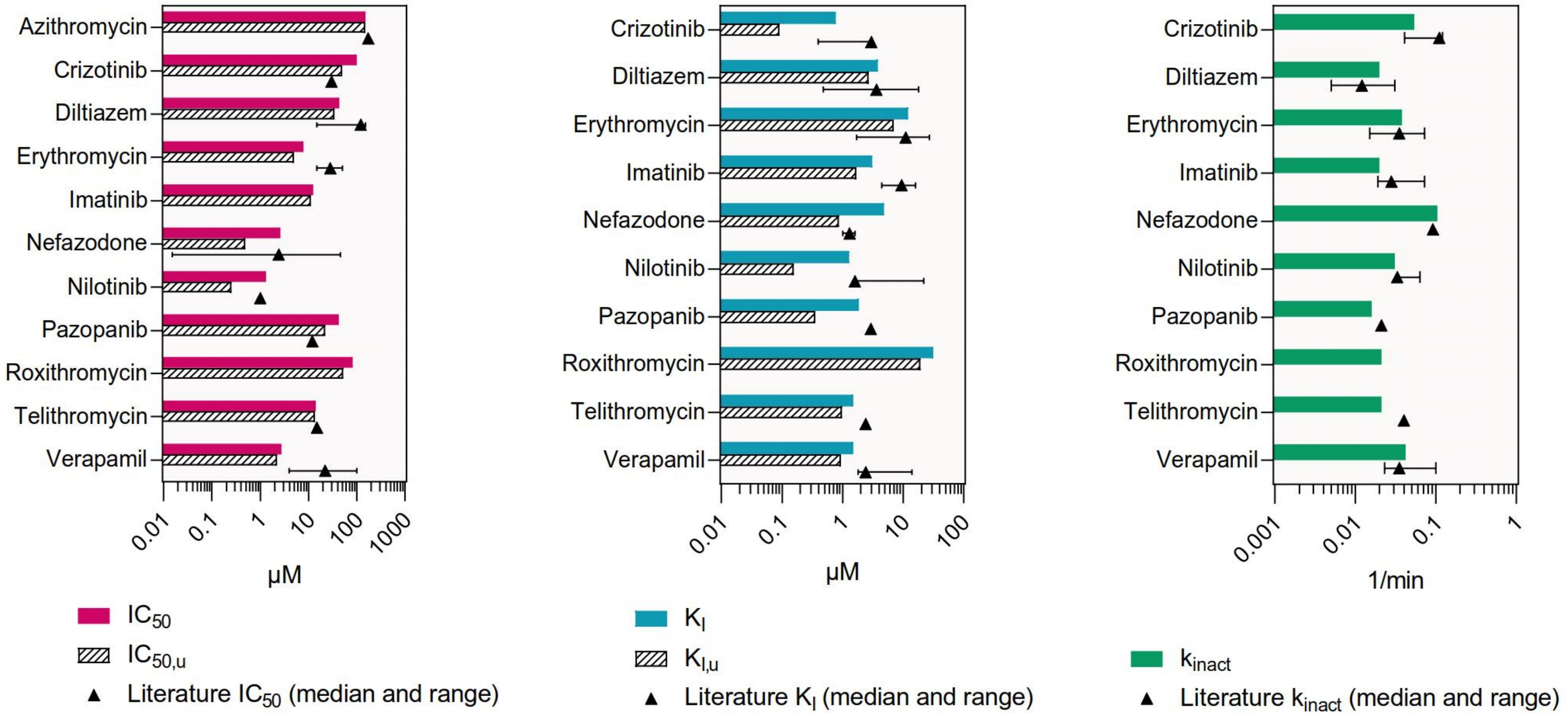

Supplementary figure S2. *Comparison of measured and literature values on CYP3A inhibition in HLM by the eleven compounds tested. The* $IC_{50,u}$ *and* $K_{I,u}$ *values have been corrected for non-specific binding to microsomal proteins (see* ***Table 1*** *in the main manuscript for exact values). Inhibition values for each inhibitor available in the DIDB Database (Copyright University of Washington 1999-2015. UW Metabolism and Transport Drug Interaction Database, accessed: February 2017) are given (median and range), telithromycin* K_I *and* k_{inact} *are from* [41]*. Similar to the measured values, the literature values shown are from HLM experiments with midazolam as the CYP3A marker substrate. No such studies have been reported for roxithromycin. No* K_I *and* k_{inact} *values were determined for azithromycin.* IC_{50}*, half maximal inhibitory concentration;* K_I*, inactivation rate constant;* k_{inact}*, maximal inactivation rate.*

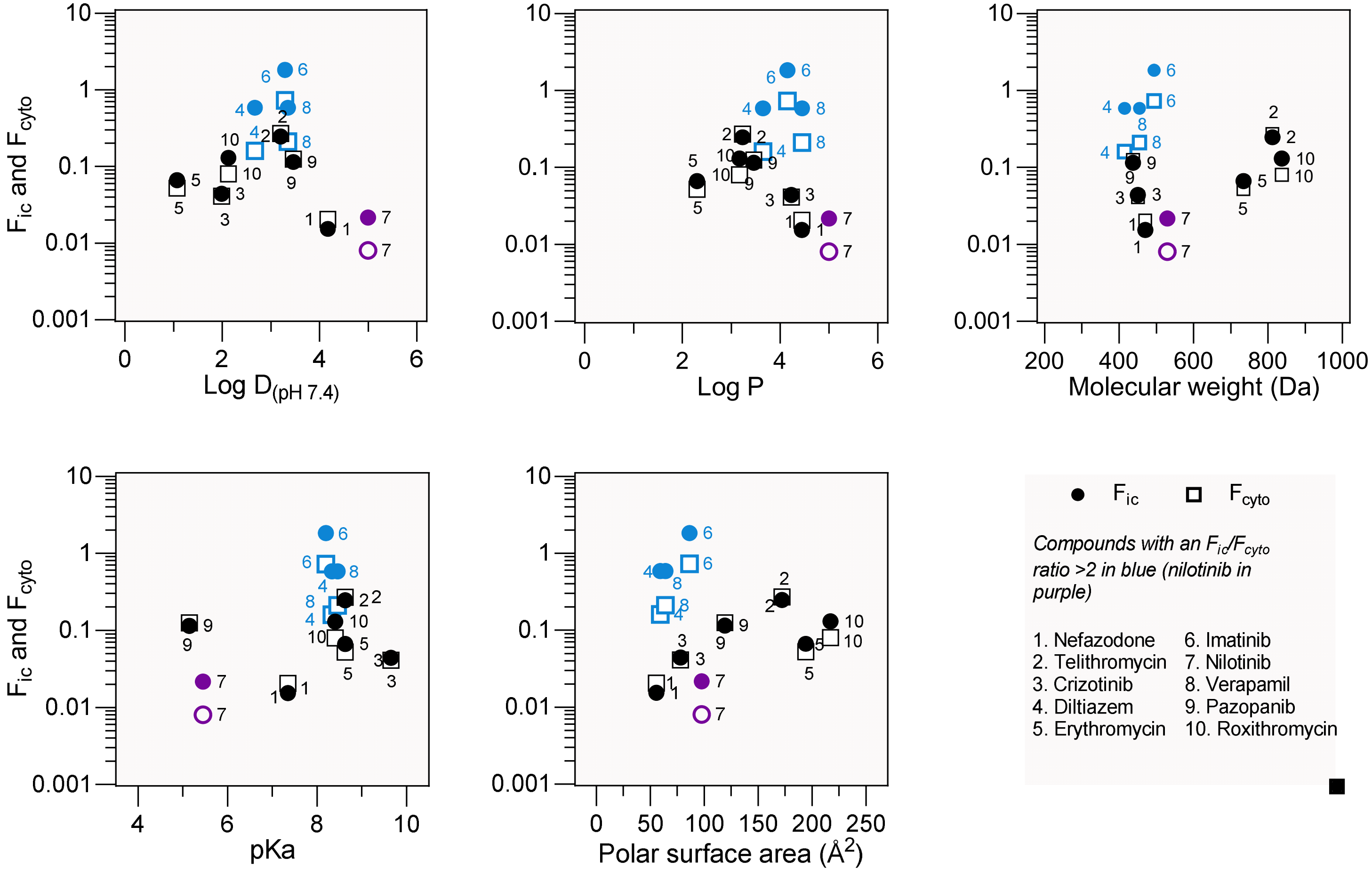

Supplementary figure S3. *Influence of physicochemical descriptors (log D, log P, molecular weight, basic pKa and polar surface area) of the tested inhibitors on their intracellular drug bioavailability (F_{ic}) and cytosolic bioavailability (F_{cyto}) in hepatocytes. Telithromycin has pKa 1 = 8.63 and pKa 2 = 1.63.*

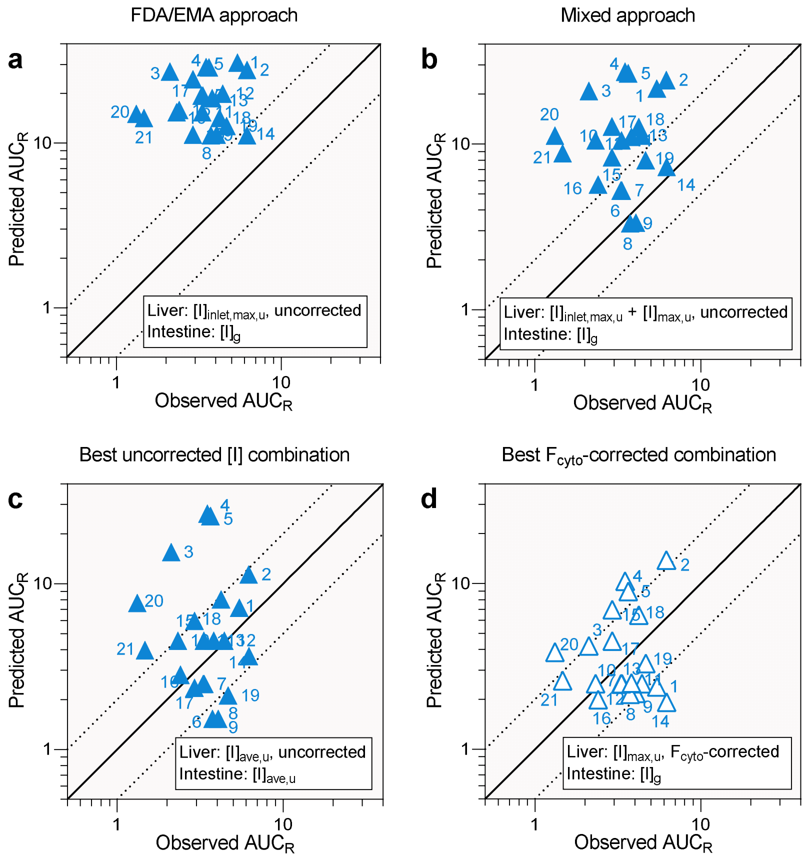

Supplementary figure S4. *Effect of F_{cyto}-correction of hepatic inhibitor concentrations on prediction accuracy. The [I] combination in **(a)** is the one recommended in drug authority guidelines, ($[I]_{inlet,max,u}$ + $[I]_g$). The [I] combination in **(b)** corresponds to a mixed term approach, ($[I]_{inlet,max,u}$ for reversible inhibition in the liver, $[I]_{max,u}$ for time-dependent inhibition in the liver, and $[I]_g$ for intestinal inhibition). Our data suggest that if no F_{cyto} values are available, $[I]_{ave,u}$ for inhibition of both hepatic and intestinal CYP3A could be used **(c)**. In the present study, the best prediction (GMFE closest to 1) was obtained with F_{cyto}-corrected $[I]_{max,u}$ in combination with $[I]_g$ **(d)**. The full line denotes no difference between predicted and observed interactions and the dashed lines indicate a prediction within two-fold of the observed values.*

*Input parameters are given in **Materials and methods** and **in Supplementary Table S5**. See **Supplementary Table S2** for detailed results of the predictions. The numbers in the subfigures refer to the individual clinical trials listed in **Supplementary Table S1**. AUC_R, the area-under-the-concentration-time curve of the substrate in the presence vs absence of the inhibitor; F_{cyto}, cytosolic bioavailability; $[I]_{ave,u}$, unbound, average inhibitor concentration; $[I]_g$, intestinal inhibitor concentration; $[I]_{inlet,max,u}$, unbound hepatic inlet inhibitor concentration based on $[I]_{max}$; $[I]_{max,u}$, unbound, peak inhibitor concentration*

References

1 FDA. U.S Food and Drug Administration, In Vitro Metabolism- and Transporter- Mediated Drug-Drug Interaction Studies Guidance for Industry. https://www.fda.gov/downloads/Drugs/GuidanceComplianceRegulatoryInformation/Guidances/UCM581965.pdf [Accessed: December 12, 2018]. (2017).

2 EMA. European Medicines Agency, Guideline on the Investigation of Drug Interactions. http://www.ema.europa.eu/docs/en_GB/document_library/Scientific_guideline/2012/07/WC500129606.pdf [Accessed: December 12, 2018]. (2012).

3 Lam, Y. W., Alfaro, C. L., Ereshefsky, L. & Miller, M. Pharmacokinetic and pharmacodynamic interactions of oral midazolam with ketoconazole, fluoxetine, fluvoxamine, and nefazodone. *J Clin Pharmacol* **43**, 1274-1282, doi:10.1177/0091270003259216 (2003).

4 NDA 021144. FDA Center for Drug Evaluation and Research. Clinical Pharmacology and Biopharmaceutics Review(s) of Telithromycin. URL: https://www.accessdata.fda.gov/drugsatfda_docs/nda/2004/21-144_Ketek.cfm (Accessed: December 12, 2018). (2004).

5 NDA 202570. FDA Center for Drug Evaluation and Research. Clinical Pharmacology and Biopharmaceutics Review(s) of Crizotinib. URL: https://www.accessdata.fda.gov/drugsatfda_docs/nda/2011/202570Orig1s000ClinPharmR.pdf (Accessed: December 12, 2018). (2011).

6 Friedman, E. J. *et al.* Effect of different durations and formulations of diltiazem on the single-dose pharmacokinetics of midazolam: how long do we go? *J Clin Pharmacol* **51**, 1561-1570, doi:10.1177/0091270010387141 (2011).

7 Backman, J. T., Olkkola, K. T., Aranko, K., Himberg, J. J. & Neuvonen, P. J. Dose of midazolam should be reduced during diltiazem and verapamil treatments. *Br J Clin Pharmacol* **37**, 221-225 (1994).

8 Okudaira, T. *et al.* Effect of the treatment period with erythromycin on cytochrome P450 3A activity in humans. *J Clin Pharmacol* **47**, 871-876, doi:10.1177/0091270007302562 (2007).

9 Olkkola, K. T. *et al.* A potentially hazardous interaction between erythromycin and midazolam. *Clin Pharmacol Ther* **53**, 298-305 (1993).

10 Zimmermann, T. *et al.* Influence of the antibiotics erythromycin and azithromycin on the pharmacokinetics and pharmacodynamics of midazolam. *Arzneimittelforschung* **46**, 213-217 (1996).

11 Kantola, T., Kivisto, K. T. & Neuvonen, P. J. Erythromycin and verapamil considerably increase serum simvastatin and simvastatin acid concentrations. *Clin Pharmacol Ther* **64**, 177-182, doi:10.1016/S0009-9236(98)90151-5 (1998).

12 O'Brien, S. G. *et al.* Effects of imatinib mesylate (STI571, Glivec) on the pharmacokinetics of simvastatin, a cytochrome p450 3A4 substrate, in patients with chronic myeloid leukaemia. *Br J Cancer* **89**, 1855-1859, doi:10.1038/sj.bjc.6601152 (2003).

13 Zhang, H. *et al.* Inhibitory effect of single and repeated doses of nilotinib on the pharmacokinetics of CYP3A substrate midazolam. *J Clin Pharmacol* **55**, 401-408, doi:10.1002/jcph.434 (2015).

14 Jacobson, T. A. Comparative pharmacokinetic interaction profiles of pravastatin, simvastatin, and atorvastatin when coadministered with cytochrome P450 inhibitors. *Am J Cardiol* **94**, 1140-1146, doi:10.1016/j.amjcard.2004.07.080 (2004).

15 Goh, B. C. *et al.* An evaluation of the drug interaction potential of pazopanib, an oral vascular endothelial growth factor receptor tyrosine kinase inhibitor, using a modified Cooperstown 5+1 cocktail in patients with advanced solid tumors. *Clin Pharmacol Ther* **88**, 652-659, doi:10.1038/clpt.2010.158 (2010).

16 Backman, J. T., Aranko, K., Himberg, J. J. & Olkkola, K. T. A pharmacokinetic interaction between roxithromycin and midazolam. *Eur J Clin Pharmacol* **46**, 551-555 (1994).

17 Yamazaki, S., Johnson, T. R. & Smith, B. J. Prediction of Drug-Drug Interactions with Crizotinib as the CYP3A Substrate Using a Physiologically Based Pharmacokinetic Model. *Drug Metab Dispos* **43**, 1417-1429, doi:10.1124/dmd.115.064618 (2015).

18 Piepho, R. W. *et al.* Pharmacokinetics of diltiazem in selected animal species and human beings. *Am J Cardiol* **49**, 525-528 (1982).

19 Idkaidek, N. M. Interplay of biopharmaceutics, biopharmaceutics drug disposition and salivary excretion classification systems. *Saudi Pharm J* **22**, 79-81, doi:10.1016/j.jsps.2013.02.002 (2014).

20 Hardman, J. G., Limbird, L.E., Gilman, A.G. in *Goodman & Gilman's The Pharmacological Basis of Therapeutics* (McGraw-Hill Professional, 2001).

21 NDA 205123. FDA Center for Drug Evaluation and Research. Clinical Pharmacology and Biopharmaceutics Review(s) of Simeprevir. URL: https://www.accessdata.fda.gov/drugsatfda_docs/nda/2013/205123Orig1s000TOC.cfm (Accessed: December 12, 2018). (2013).

22 Kretz, O., Weiss, H. M., Schumacher, M. M. & Gross, G. In vitro blood distribution and plasma protein binding of the tyrosine kinase inhibitor imatinib and its active metabolite, CGP74588, in rat, mouse, dog, monkey, healthy humans and patients with acute lymphatic leukaemia. *Br J Clin Pharmacol* **58**, 212-216, doi:10.1111/j.1365-2125.2004.02117.x (2004).

23 Peng, B., Lloyd, P. & Schran, H. Clinical pharmacokinetics of imatinib. *Clin Pharmacokinet* **44**, 879-894, doi:10.2165/00003088-200544090-00001 (2005).

24 Peng, B. *et al.* Pharmacokinetics and pharmacodynamics of imatinib in a phase I trial with chronic myeloid leukemia patients. *J Clin Oncol* **22**, 935-942, doi:10.1200/JCO.2004.03.050 (2004).

25 Petain, A. *et al.* Population pharmacokinetics and pharmacogenetics of imatinib in children and adults. *Clin Cancer Res* **14**, 7102-7109, doi:10.1158/1078-0432.CCR-08-0950 (2008).

26 Mao, J., Mohutsky, M. A., Harrelson, J. P., Wrighton, S. A. & Hall, S. D. Predictions of cytochrome P450-mediated drug-drug interactions using cryopreserved human hepatocytes: comparison of plasma and protein-free media incubation conditions. *Drug Metab Dispos* **40**, 706-716, doi:10.1124/dmd.111.043158 (2012).

27 Barbhaiya, R. H., Dandekar, K. A. & Greene, D. S. Pharmacokinetics, absolute bioavailability, and disposition of [14C]nefazodone in humans. *Drug Metab Dispos* **24**, 91-95 (1996).

28 Greene, D. S., Salazar, D. E., Dockens, R. C., Kroboth, P. & Barbhaiya, R. H. Coadministration of nefazodone and benzodiazepines: III. A pharmacokinetic interaction study with alprazolam. *J Clin Psychopharmacol* **15**, 399-408 (1995).

29 Kenny, J. R. *et al.* Drug-drug interaction potential of marketed oncology drugs: in vitro assessment of time-dependent cytochrome P450 inhibition, reactive metabolite formation and drug-drug interaction prediction. *Pharm Res* **29**, 1960-1976, doi:10.1007/s11095-012-0724-6 (2012).

30 Larson, R. A. *et al.* Population pharmacokinetic and exposure-response analysis of nilotinib in patients with newly diagnosed Ph+ chronic myeloid leukemia in chronic phase. *Eur J Clin Pharmacol* **68**, 723-733, doi:10.1007/s00228-011-1200-7 (2012).

31 Hurwitz, H. I. *et al.* Phase I trial of pazopanib in patients with advanced cancer. *Clin Cancer Res* **15**, 4220-4227, doi:10.1158/1078-0432.CCR-08-2740 (2009).

32 Lassman, H. B., Puri, S. K., Ho, I., Sabo, R. & Mezzino, M. J. Pharmacokinetics of roxithromycin (RU 965). *J Clin Pharmacol* **28**, 141-152 (1988).

33 Halstenson, C. E. *et al.* Disposition of roxithromycin in patients with normal and severely impaired renal function. *Antimicrob Agents Chemother* **34**, 385-389 (1990).

34 Zhao, P. *et al.* Evaluation of exposure change of nonrenally eliminated drugs in patients with chronic kidney disease using physiologically based pharmacokinetic modeling and simulation. *J Clin Pharmacol* **52**, 91S-108S, doi:10.1177/0091270011415528 (2012).

35 Rowland Yeo, K., Walsky, R. L., Jamei, M., Rostami-Hodjegan, A. & Tucker, G. T. Prediction of time-dependent CYP3A4 drug-drug interactions by physiologically based pharmacokinetic modelling: impact of inactivation parameters and enzyme turnover. *Eur J Pharm Sci* **43**, 160-173, doi:10.1016/j.ejps.2011.04.008 (2011).

36 Keefe, D. L., Yee, Y. G. & Kates, R. E. Verapamil protein binding in patients and in normal subjects. *Clin Pharmacol Ther* **29**, 21-26 (1981).

37 John, D. N., Fort, S., Lewis, M. J. & Luscombe, D. K. Pharmacokinetics and pharmacodynamics of verapamil following sublingual and oral administration to healthy volunteers. *Br J Clin Pharmacol* **33**, 623-627 (1992).

38 Belz, G. G., Doering, W., Munkes, R. & Matthews, J. Interaction between digoxin and calcium antagonists and antiarrhythmic drugs. *Clin Pharmacol Ther* **33**, 410-417 (1983).

39 Kerr, D. J. *et al.* The effect of verapamil on the pharmacokinetics of adriamycin. *Cancer Chemother Pharmacol* **18**, 239-242 (1986).

40 Anderson, P., Bondesson, U. & de Faire, U. Pharmacokinetics of verapamil in patients with hypertension. *Eur J Clin Pharmacol* **31**, 155-163 (1986).

41 Magee, T. V. *et al.* Discovery of azetidinyl ketolides for the treatment of susceptible and multidrug resistant community-acquired respiratory tract infections. *J Med Chem* **52**, 7446-7457, doi:10.1021/jm900729s (2009).
